# Differential regulation of microglial states by colony stimulating factors

**DOI:** 10.3389/fncel.2023.1275935

**Published:** 2023-10-30

**Authors:** E. Richard Stanley, Fabrizio Biundo, Şölen Gökhan, Violeta Chitu

**Affiliations:** ^1^Department of Developmental and Molecular Biology, Albert Einstein College of Medicine, Bronx, NY, United States; ^2^Department of Neurology, Albert Einstein College of Medicine, Institute for Brain Disorders and Neural Regeneration, Bronx, NY, United States

**Keywords:** CSF-1, CSF-2, CSF-3, CSF-1 receptor, microglia, demyelinating disease, CRL, ALSP

## Abstract

Recent studies have emphasized the role of microglia in the progression of many neurodegenerative diseases. The colony stimulating factors, CSF-1 (M-CSF), granulocyte-macrophage CSF (GM-CSF) and granulocyte CSF (G-CSF) regulate microglia through different cognate receptors. While the receptors for GM-CSF (GM-CSFR) and G-CSF (G-CSFR) are specific for their ligands, CSF-1 shares its receptor, the CSF-1 receptor-tyrosine kinase (CSF-1R), with interleukin-34 (IL-34). All four cytokines are expressed locally in the CNS. Activation of the CSF-1R in macrophages is anti-inflammatory. In contrast, the actions of GM-CSF and G-CSF elicit different activated states. We here review the roles of each of these cytokines in the CNS and how they contribute to the development of disease in a mouse model of CSF-1R-related leukodystrophy. Understanding their roles in this model may illuminate their contribution to the development or exacerbation of other neurodegenerative diseases.

## 1. Regulation of microglia by colony stimulating factors

Microglia are the macrophages of the brain. They comprise ~10% of the brain cells and are distributed throughout the central nervous system (CNS) (Lawson et al., 1990; Yang et al., 2013) [reviewed in Ginhoux and Prinz (2015)]. Their CNS-specific roles include the maintenance of brain homeostasis and the modulation of neural circuits (Paolicelli et al., 2011; Parkhurst et al., 2013; Ueno et al., 2013; Wake et al., 2013). In adult healthy brain, they exhibit a ramified morphology, surveying their surrounding area (Davalos et al., 2005; Nimmerjahn et al., 2005; Hanisch and Kettenmann, 2007). In neuronal injury and in neurodegenerative diseases, microglia become activated and adopt a hypertrophic or ameboid shape (Lobsiger et al., 2013; Roth et al., 2015; Fernandez-Arjona et al., 2017). Activated microglia may have opposing functions (David and Kroner, 2011; Hu et al., 2015). They can secrete neurotrophic factors to protect damaged neurons and phagocytose cellular debris, permitting tissue regeneration (Nakajima and Kohsaka, 2004; Neumann et al., 2009). Alternatively, when excessively activated, they can secrete neurotoxic molecules, such as reactive nitrogen and oxygen species (NOS and ROS)(Block et al., 2007; Brown and Neher, 2014; Fu et al., 2014).

CSF-1R, GM-CSFR and G-CSFR are all expressed on microglia. While their expression has been reported on some neural lineage cells [reviewed in Chitu et al. (2021)], our focus here is on the actions of the CSFs mediated via their microglial receptors.

### 1.1. Systemic and local expression of CSFs

In blood, CSF-1 circulates at physiological concentrations of 4.5 ng/mL (Bartocci et al., 1987; Janowska-Wieczorek et al., 1991), while circulating IL-34 concentrations (52 pg./mL) are substantially lower (Wang et al., 2016). Circulating GM-CSF (Kadar et al., 1997; Frydecka et al., 2018) and G-CSF (Kiriyama et al., 1993; Ishiguro et al., 1996; Laske et al., 2009) are normally barely detectable, but rise in response to inflammatory stimuli [reviewed in Dougan et al. (2019), Hamilton (2020)]. In humans, circulating GM-CSF gradually increases with age (Tarkowski et al., 2001), while G-CSF decreases (Ishiguro et al., 1996; Laske et al., 2009). At normal physiological concentrations, recombinant CSF-1 fails to cross the blood–brain barrier (BBB) (Chitu and Stanley, unpublished). In contrast, GM-CSF can readily penetrate the BBB (McLay et al., 1997). In rodents, G-CSF was also reported to slowly cross the BBB (Zhao et al., 2007). However, in humans its transport is limited (Yuan et al., 2015; Yoshio et al., 2016).

In normal brain, CSF-1 and IL-34 are primarily expressed by CNS neurons, in a largely non-overlapping manner (Greter et al., 2012; Nandi et al., 2012; Wang et al., 2012). CSF-1 is also expressed by glia (Lee et al., 1993; Thery and Mallat, 1993) and at low levels by microglia (Walker et al., 2017). The regional expression of CSF-1 and IL-34 is also largely non-overlapping, with CSF-1 being found primarily in the cerebellum and white matter and IL-34 in the forebrain and grey matter (Wei et al., 2010; Easley-Neal et al., 2019; Kana et al., 2019). GM-CSF (encoded by the *Csf2* gene) and G-CSF (encoded by the *Csf3* gene) are also expressed in brain at steady state, but at low levels [reviewed in Chitu et al. (2021) and Biundo et al. (2023b)], suggesting important actions at low concentrations and/or paracrine signaling *via* local production and utilization.

### 1.2. CSF-1R signaling is required for the development, maintenance, and homeostatic functions of CNS microglia

In the steady state, macrophage survival and proliferation are primarily regulated by the CSF-1R *via* both circulating and locally expressed CSF-1R ligands that regulate macrophage development and maintain tissue macrophage densities (Tushinski et al., 1982; Bartocci et al., 1987; Cecchini et al., 1994; Ryan et al., 2001). Consistent with these primarily trophic and anti-inflammatory roles (Cecchini et al., 1994; Chitu and Stanley, 2006), CSF-1 induces miRNA-21 expression in macrophages that suppresses their expression of inflammatory mediators and enhances anti-inflammatory marker expression (Caescu et al., 2015).

The CSF-1R plays a central role in the development of microglia from yolk sac progenitors as well as their maintenance in adult life. In mice, erythro-myeloid progenitors (EMPs) in the yolk sac give rise to microglia and meningeal, perivascular and some choroid plexus macrophages in a CSF-1R-dependent manner (Ginhoux et al., 2010; Hoeffel et al., 2012; Munro et al., 2020) [reviewed in Prinz et al. (2017)]. CSF-1R expression is first apparent at E8 in EMPs (Gomez Perdiguero et al., 2015) and at E9 in EMP-derived A2 progenitors (Kierdorf et al., 2013). Following the development of the fetal circulatory system, the A2 progenitors colonize the developing brain and spinal cord at ~E9.5, where they give rise to microglia (Ginhoux et al., 2010; Schulz et al., 2012; Kierdorf et al., 2013; Hagemeyer et al., 2016) and the other macrophages (Goldmann et al., 2016). BBB development at ~E11.5 prevents contribution from hematopoietic stem cell-derived monocytes to the establishment of parenchymal microglia (Ginhoux et al., 2010). During brain development, CSF-1R signaling also promotes the postnatal colonization of the subventricular zone by microglia (Lelli et al., 2013) and the establishment of microglial processes (Wegiel et al., 1998; Sasaki et al., 2000).

Regulation by the CSF-1R ligands differs temporally and spatially. Temporally, CSF-1 alone is required for microglial colonization and maintenance in fetal brain, while IL-34 begins to be required postnatally (Greter et al., 2012; Nandi et al., 2012). Spatially, cerebellar microglia are uniquely dependent on CSF-1 for their development and maintenance, whereas forebrain microglia mainly require IL-34 (Kana et al., 2019). Further differential dependence on CSF-1R ligands is observed in the forebrain, where the white matter microglia are regulated by CSF-1 and the grey matter microglia by IL-34 (Easley-Neal et al., 2019; Badimon et al., 2020).

Inhibition of CSF-1R signaling in the adult brain leads to massive (90–99%) microglial death (Elmore et al., 2014; Bruttger et al., 2015; Huang et al., 2018). Following cessation of treatment with CSF-1R inhibitors, the restoration of the microglial population is contributed to by the proliferation of microglia resistant to CSF-1R inhibition that share transcriptional profile similarities with microglial progenitors in the yolk sac (Zhan et al., 2020; Hohsfield et al., 2021). It is unclear which receptors provide survival signals for these resistant cells. A pathway involving autocrine MAC2/TREM2-TYROBP has been suggested to contribute (Zhan et al., 2020). In addition, the finding that following interactions with apoptotic neurons, subsets of microglia lose their dependence on CSF-1R signaling for survival, which in turn is maintained via the receptor tyrosine kinase Axl (Anderson et al., 2022), suggests another possible mechanism.

**Figure 1:**
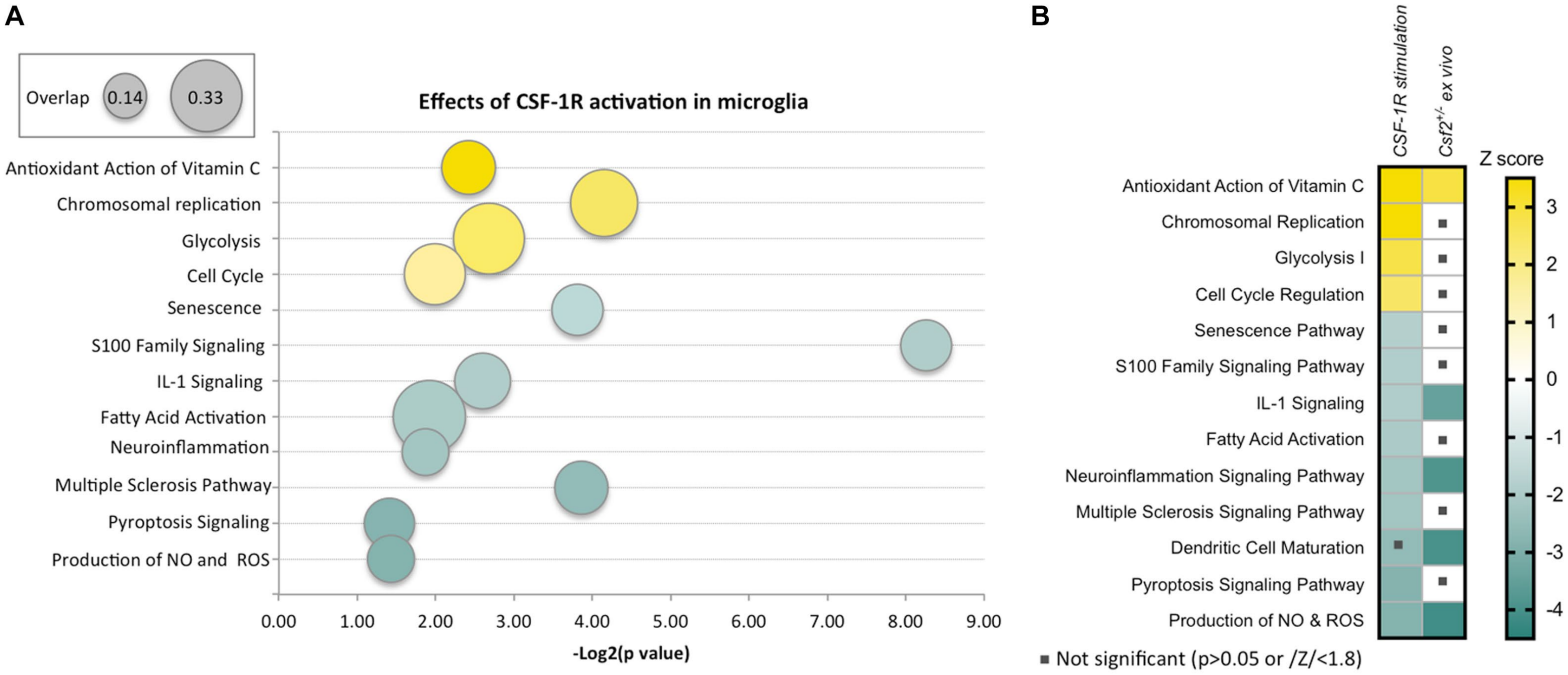
Effects of CSF-1R activation in microglia and overlap with the effects of reduced GM-CSF signaling. **(A)** Ingenuity Pathway Analysis-generated representation of pathways activated (yellow) or inhibited (green) in primate microglia following cultivation in CSF-1-containing medium compared to *ex vivo* microglia. The figure was generated using a published data set (Timmerman et al., 2022). The ratio of number of differentially expressed genes to the total number of genes in the pathway is indicated by the size of the bubble. The sizes corresponding to the minimum (0.14) and maximum (0.33) overlap ratios are indicated in the top left box. **(B)** Decreased GM-CSF bioavailability *in vivo* induces a microglial state that partially overlaps with chronic CSF-1R stimulation *in vitro*. The heatmap shows a comparison of the pathways represented in **(A)** to pathways dysregulated in microglia isolated from *Csf2^+/−^* mice compared to wt microglia (Chitu et al., 2020). The *Z*-score scale shown on the right applies to both panels. Note that the overlapping pathways are increased antioxidant action of vitamin C and decreased IL-1 signaling, neuroinflammation signaling, dendritic cell maturation and production of NO and ROS.

*In vitro*, CSF-1 induces the proliferation of murine (Suzumura et al., 1990) and human (Lee et al., 1994) microglia. Two recent *ex vivo* studies utilizing human or *Macaca mulatta* microglia show that their culture in either CSF-1 or IL-34 induces identical transcriptional responses (Walker et al., 2017; Timmerman et al., 2022). Pathway analysis of the latter dataset indicates that activation of CSF-1R suppresses senescence and inflammatory pathways, including the production of NOS and ROS and activates metabolic and antioxidant pathways (Timmerman et al., 2022) (Figure 1A). These data are consistent with a role of CSF-1R signaling in maintaining a homeostatic phenotype in microglia.

### 1.3. Regulation of microglial functions by GM-CSF

Reduction of GM-CSF expression leads to decreased cognitive function (Krieger et al., 2012; Chitu et al., 2020), indicating that GM-CSF signaling has an important role in the functioning of the nervous system in the steady state. Intriguingly, the two subunits of GM-CSF receptor, the low affinity chain GMRα (encoded by *Csf2ra*) and the high affinity βc (encoded by *Csf2rb*), exhibit different patterns of expression in the brain, with *Csf2ra* transcripts being expressed both in microglia and neural lineage cells, and *Csf2rb* transcripts being largely restricted to microglia and having sporadic expression in the neural lineage.^1^ Functional studies revealed the presence of high affinity GM-CSFRs on primary rat oligodendrocytes (Baldwin et al., 1993) and in microglia, while the expression of GM-CSFRs in astrocytes is controversial [reviewed in Chitu et al. (2021)]. However, since the GMRα subunit alone can independently signal for glucose transport (Ding et al., 1994), the absence of βc in some cell types does not imply a lack of response to GM-CSF.

In cultured microglia, activation of GM-CSFRs induces a series of responses including rapid proliferation (Giulian and Ingeman, 1988; Suzumura et al., 1990; Ganter et al., 1992; Lee et al., 1994; Suh et al., 2005), development of ramified processes (Tanaka et al., 1997) and a series of gene expression changes. These include the upregulation of both *Csf2ra* and *Csf2rb* and the downregulation of *Csf1r* transcripts (Re et al., 2002), suggestive of antagonistic roles of the two receptors in the control of microglia function. Indeed, while CSF-1 suppresses (Lee et al., 1993), GM-CSF induces MHC II expression in rodent microglia as well as the expression of co-stimulatory molecules B7-1 (CD80) and CD24 (Matyszak et al., 1999; Wei and Jonakait, 1999; Re et al., 2002) and enhances their T cell stimulatory function (Aloisi et al., 2000; Re et al., 2002). However, a recent report shows that GM-CSF does not increase the expression of HLA-DR, HLA-ABC or CD80 in human microglia (Farzam-Kia et al., 2023). It remains to be established whether the priming of antigen presentation by GM-CSF is species-specific. While GM-CSF alone does not induce the secretion of the classical inflammatory cytokines IL-1β or TNFα, it has been reported to induce IL-6 production by cultured microglia (Suzumura et al., 1990; Farzam-Kia et al., 2023). This effect was not reproduced in organotypic hippocampal cultures (Dikmen et al., 2020), suggesting that *in situ* microglia might receive additional inputs that counteract the effect of GM-CSF. Nevertheless, even in the absence of inflammatory activation, GM-CSF induced a microglial state that caused long-term disruption of neuronal network rhythms in organotypic cultures (Dikmen et al., 2020) and behavioral deficits consistent with schizophrenia after intra-hippocampal administration *in vivo* (Zhu et al., 2014).

GM-CSF also increases the proteolytic activity of microglia through stimulation of Cathepsin S secretion (Liuzzo et al., 1999) and upregulation of the expression of Cathepsin F, MMP-11, and MMP-12 (Re et al., 2002). Both myelin basic protein (MBP) and the myelin oligodendrocyte glycoprotein (MOG) are Cathepsin S substrates (Haves-Zburof et al., 2011) and MMP-12 is also involved in the degradation of myelin (Hansmann et al., 2012). Since GM-CSF was also reported to increase the ability of microglia to phagocytose myelin (Smith, 1993) it is tempting to speculate that GM-CSF-activated microglia might play a role in demyelinating diseases. Interestingly, GM-CSF was also reported to increase microglial oxidative activity *in vitro* (Smith et al., 1998; Tambuyzer and Nouwen, 2005) and to prevent the downregulation of microglial immune functions (TNFα secretion, antigen presentation) after ingestion of apoptotic cells (Magnus et al., 2004). Consistent with these observations, *ex vivo* transcriptomic profiling of microglia isolated from *Csf2^+/−^* mice reveals activation of antioxidant pathways and suppression of microglia activation, dendritic maturation and neuroinflammation (Figure 1B) (Chitu et al., 2020). Together, these data suggest that although GM-CSF does not promote an overt inflammatory reaction, it disrupts the physiological functions of microglia to prime responses associated with tissue injury.

### 1.4. Regulation of microglial functions by G-CSF

*Csf3-*deficient mice have impaired memory and motor deficits, decreased adult neurogenesis in the dentate gyrus area of the hippocampus, a long-term potentiation deficit and reduced dendritic complexity of hippocampal neurons (Diederich et al., 2009), indicating that G-CSF signaling in the CNS also plays important roles in the steady state. G-CSF is neuronally expressed and is co-expressed with the G-CSFR in cortical neurons, where it has been suggested that its autocrine action stimulates neuronal survival (Schneider et al., 2005). However, single cell transcriptome profiling of the mouse and human brains shows that *Csf3r* transcripts encoding the G-CSFR are found mainly in microglia (databases https://www.proteinatlas.org/, http://dropviz.org/, www.microgliasinglecell.com, https://singlecell.broadinstitute.org/single_cell/study/SCP795/a-transcriptomic-atlas-of-the-mouse-cerebellum and https://portals.broadinstitute.org/single_cell/study/aging-mouse-brain) (Saunders et al., 2018; Hammond et al., 2019; Ximerakis et al., 2019; Kozareva et al., 2021). Despite this, information regarding the effects of G-CSF on microglial activity is limited. While G-CSF is not a microglial mitogen (Giulian and Ingeman, 1988), its administration was reported to increase microglial chemotaxis (Yamasaki et al., 2010), to activate a Cathepsin S-CX3CR1-inducible NOS pathway and to induce the production of factors that promote neuronal excitability (Basso et al., 2017). Together, these data suggest that many of the effects reported following G-CSF administration *in vivo* [reviewed in Chitu et al. (2021)] might be mediated through regulation of microglia function.

### 1.5. Studies in *Csf1r^+/−^* mice suggest that imbalanced colony stimulating factor signaling leads to dyshomeostatic microglia

Treatment with CSF-1R inhibitors (Elmore et al., 2014), or targeted inactivation of the *Csf1r* (Ginhoux et al., 2010; Bruttger et al., 2015) lead to microglial death and systemically administered CSF-1 fails to cross the BBB. Thus, analysis of the role of CSF-1R in microglia function has been limited. However, *Csf1r* heterozygosity reduces the level of cell surface CSF-1Rs without causing depletion of microglia (Chitu et al., 2015; Biundo et al., 2023a) thus offering a unique opportunity to determine how reduced CSF-1R signaling impacts microglial function. Studies to date indicate that reduced CSF-1R signaling causes a dyshomeostatic microglial phenotype (Chitu et al., 2020; Kempthorne et al., 2020; Arreola et al., 2021; Biundo et al., 2021, 2023b) that is in part mediated by increased expression of GM-CSF and G-CSF. The following section discusses the *Csf1r^+/−^* model of CSF-1 receptor-related leukodystrophy and how it can contribute to our understanding of the role of growth factor and cytokine receptors in demyelinating disease.

## 2. Role of colony stimulating factors in CSF-1 receptor-related leukodystrophy

### 2.1. CSF-1 receptor-related leukodystrophy

Rademakers and colleagues were the first to show that the adult-onset hereditary leukoencephalopathy known as Hereditary Diffuse Leukoencephalopathy with Spheroids (HDLS) was caused by dominantly inherited, mono-allelic, *CSF1R* mutations (Rademakers et al., 2011). This disease, known previously by several other names [reviewed in Chitu et al. (2021)] and now named CSF1R-related leukoencephalopathy (CRL) (OMIM #221820), is associated with cognitive impairment, psychiatric disorders, motor dysfunction and seizures, with an average onset at approximately 43 years of age (Konno et al., 2017b). Over 100 different *CSF1R* mutations have been reported in CRL patients, the vast majority being missense mutations within the tyrosine kinase domain. Three of these mutations cause nonsense-mediated mRNA decay (NMD) and are therefore expected to result in haploinsufficiency (Konno et al., 2014b; Miura et al., 2018; Leng et al., 2019). No clear genotype–phenotype correlation is apparent, apart from an earlier average disease onset in patients carrying truncating mutations or those that trigger NMD (Chitu et al., 2022). The penetrance of CRL is age-dependent, increasing from 10% at age 27 years, to 50% at age 43 years and reaching 95% at age 60 years. However, familial case reports, identifying healthy carriers of multiple different *CSF1R* mutations that are pathogenic in their close relatives [reviewed in Chitu et al. (2022)], suggest incomplete penetrance even in aged individuals. Thirty-four *CSF1R* pathogenic mutations reported in CRL have been characterized in terms of expression and signaling (Hiyoshi et al., 2013; Nicholson et al., 2013; Konno et al., 2014b; Miura et al., 2018; Kraya et al., 2019; Tian et al., 2019; Delaney et al., 2020). Apart from the 3 NMD mutations, the remaining missense mutations, with one possible exception, are loss of function [reviewed in Chitu et al. (2022)]. Although some authors have suggested that CRL mutations cause a dominant-negative, inhibitory function of the mutant chain (Hume et al., 2020), co-transfection experiments of wild type and mutant *CSF1R* constructs showed that some mutant chains do not significantly inhibit the phosphorylation of wild type CSF-1R in the response to CSF-1 (Konno et al., 2014a; Delaney et al., 2020), indicating that a dominant-negative mechanism is unlikely. Thus, while some of the uncharacterized CRL mutations may exhibit dominant-negative functions, haploinsufficiency is an underlying mechanism as evidenced by the 3 NMD mutations.

The most common initial complaint of CRL patients is cognitive impairment followed by gait disorders and dyskinesia which tend to be more common in females [reviewed in Chitu et al. (2022)]. These symptoms are associated with structural alterations in white matter that usually involve the corpus callosum. Histopathologically, the defining features of CRL are the presence of lipid-laden pigmented phagocytes in the affected white matter and of axonal dilations (spheroids) indicative of neurodegeneration. Interestingly, the development of spheroids is paralleled by microglial activation and there is suggestive evidence for a contribution of microglial-generated oxidative stress to the neuronal pathology and demyelination [reviewed in Chitu et al. (2022)].

### 2.2. The heterozygous *Csf1r* mouse model of CRL

#### 2.2.1. Rationale for the Csf1r heterozygous mouse model

The development of the *Csf1r* heterozygous C57BL6/J mouse as a model of CRL was catalyzed by the first description of *CSF1R* haploinsufficiency as a cause of CRL (Konno et al., 2014a) and supported by subsequent reports of two additional CRL mutations that cause NMD (Miura et al., 2018, Leng et al., 2019). The C57BL6/J background was chosen for two reasons: Firstly, this background caused a more severe *Csf1r* deficiency phenotype than the original FVB/NJ background and was therefore more likely to yield disease in *Csf1r* heterozygotes. Secondly, because of the availability of numerous mutant and transgenic lines on this background that would facilitate investigation of the underlying mechanisms of CRL development.

#### 2.2.2. Characteristics of disease in the mouse model

*Csf1r^+/−^* mice develop cognitive deficits, motor coordination deficits (more marked in females), depression- and anxiety- like behavior (Chitu et al., 2015, 2020; Biundo et al., 2021), that are characteristic of CRL (Konno et al., 2018) [reviewed in Chitu et al. (2022)]. The penetrance of disease in 15-month-old mice is 60–70% (Chitu et al., 2022, 2023), corresponding to a similar degree of penetrance in humans at the equivalent age (43 years) (Konno et al., 2017b; Wang et al., 2020). The course of disease progression in mice is accelerated by a higher fat-containing diet [reviewed in Chitu et al. (2022)]. Magnetic resonance imaging consistently shows callosal atrophy without calcifications. Decreased myelin staining, increases in the G-ratio of myelinated axons and in Cystatin 7 expression, indicative of active demyelination coupled with remyelination, together with axonal degeneration and the presence of axonal spheroids, are observed in the mouse model (Chitu et al., 2020; Biundo et al., 2021).

*Csf1r^+/−^* mice exhibit slightly increased microglial densities throughout the brain, even in young asymptomatic mice (Chitu et al., 2015; Arreola et al., 2021), that are secondary to elevation of cerebral *Csf2* expression (Chitu et al., 2020). In symptomatic *Csf1r^+/−^* mice, microglia are unevenly distributed in the white matter, with occasional clustering in the periventricular area of the corpus callosum and sporadically in the cerebellar white matter (Chitu et al., 2020; Biundo et al., 2021). Clustering of white matter microglia, which is also observed in CRL (Chitu et al., 2020) has recently been correlated with the active clearing of degenerated myelin (Safaiyan et al., 2021) and might reflect active pathology. Within the corpus callosum, the microglia exhibit reduced ramification, consistent with an activated phenotype similar to that reported in early (Stage II) CRL (Oyanagi et al., 2017). Also similar to CRL (Ali et al., 2007; Kawakami et al., 2016; Oyanagi et al., 2017), there is increased GFAP staining, indicative of astrocytosis, specifically within the areas of microgliosis (Chitu et al., 2015; Biundo et al., 2021). *Csf1r^+/−^* mice also exhibit a decrease in the density of NeuN^+^ neurons in cortical Layer V that may be related to the callosal thinning, since some neurons within this layer project to the corpus callosum (Ivy and Killackey, 1981). Overall, the available data indicate that symptomatic 18-month-old *Csf1r^+/−^* mice exhibit behavioral deficits similar to those found in CRL patients, accompanied by histological features similar to early stages (Stage II) of CRL [reviewed in greater detail in Chitu et al. (2022)].

#### 2.2.3. Evidence that CRL is a primary microgliopathy

Detailed pathological analysis of microglia in CRL (Oyanagi et al., 2017) led to the concept that CRL is a primary microgliopathy in which a genetic defect linked to microglia causes neurodegeneration (Konno et al., 2018). Given the reported expression of the CSF-1R in neuronal cell populations (Wang et al., 1999; Nandi et al., 2012; Luo et al., 2013; Clare et al., 2018), we addressed the contribution of *Csf1r* heterozygosity in the microglial and neuronal lineages to CRL development in the mouse model by studying disease development in *Csf1r^fl/+^; Cx3cr1^Cre/+^* and *Csf1r^fl/+^; Nestin^Cre/+^* mice, in which one allele of the *Csf1r* was deleted in the mononuclear phagocytes and neural lineages, respectively (Biundo et al., 2021). These studies showed that *Csf1r* heterozygosity in the mononuclear phagocytic lineage was sufficient to reproduce all aspects of the neurodegenerative disease of *Csf1r^+/−^* mice, with no discernable effect of deletion in the neural lineage. Furthermore, an independent study showed that there was no increased infiltration by peripheral leukocytes into the brains of symptomatic *Csf1r^+/−^* mice (Chitu et al., 2020). Together, these data indicate that in the mouse, CRL is a primary microgliopathy.

#### 2.2.4. Roles of GM-CSF and G-CSF in CRL

Early studies of the *Csf1r^+/−^* mouse model revealed that despite decreased expression of the CSF-1R, microglial densities were unexpectedly slightly elevated, both prior to, and after, disease development (Chitu et al., 2015; Arreola et al., 2021). This increase in microglia was not due to a compensatory increase in either of the CSF-1R ligands, CSF-1 or IL-34, as neither their mRNA, nor protein levels were altered (Chitu et al., 2015). Thus, to determine how this increased microglial density might arise on the background of decreased CSF-1R expression, mRNAs of approximately 40 inflammatory cytokines, chemokines and receptors were screened. Of these, only *Csf2* and *Csf3* mRNAs were elevated in the brains of 7-week-old pre-symptomatic *Csf1r^+/−^* mice and remained elevated in the older symptomatic mice (Chitu et al., 2015). These results prompted investigations with large cohorts of wt, single, and double mutant (*Csf1r^+/−^, Csf1r^+/−^; Csf2^+/−^, Csf1r^+/−^; Csf3^+/^, ^−^Csf2^+/−^* and *Csf3^+/−^*) male and female mice (Chitu et al., 2020; Biundo et al., 2023b), discussed below. In the latter studies, both *CSF2* and *CSF3* mRNAs were also shown to be elevated in the brains of post-mortem CRL patients and alterations in CSF-3 signaling were also noted in an independent study (Kempthorne et al., 2020).

#### 2.2.5. Role of GM-CSF and G-CSF in the development of behavioral deficits in the *Csf1r^+/−^* model of CRL

Starting from 7 months of age, *Csf1r^+/−^* mice progressively develop cognitive and sensorimotor deficits that are associated with the loss of callosal white matter (Chitu et al., 2015, 2020; Li et al., 2023). By connecting the cerebral hemispheres, the corpus callosum facilitates the integration and processing of motor, sensory, and cognitive signals. Thus, disruption of myelination could potentially contribute to both types of deficit. Consistent with the studies summarized in Section 1.3, which suggest that GM-CSF promotes a demyelinating state in microglia, examination of *Csf1r^+/−^; Csf2^+/−^* mice revealed a substantial contribution of GM-CSF to microglia activation and demyelination in the callosal white matter (Chitu et al., 2020). Improvement of myelination in *Csf1r^+/−^; Csf2^+/−^* mice was associated with attenuation of both cognitive and motor deficits. In contrast, targeting G-CSF did not improve myelination and had no effect on cognition. However, monoallelic disruption of *Csf3* selectively rescued the motor coordination deficits of female *Csf1r^+/−^* mice, while it also tended to worsen motor function in males (Biundo et al., 2023b). This improvement might be related to attenuation of microglia dyshomeostasis in the cerebellum. Interestingly, ataxia and cerebellar involvement have been reported predominantly in female CRL patients (Mateen et al., 2010; Guerreiro et al., 2013; Karle et al., 2013; Riku et al., 2014; Kim et al., 2015, 2019; Meyer-Ohlendorf et al., 2015; Sundal et al., 2015; Lynch et al., 2016, 2017; Bonvegna et al., 2020). Together, these data suggest that GM-CSF plays a major role in the development of CRL-like disease, while G-CSF might contribute to sex-specific phenotypes.

#### 2.2.6. Role of GM-CSF and G-CSF in microglial dyshomeostasis in the *Csf1r^+/−^* model of CRL

**Figure 2:**
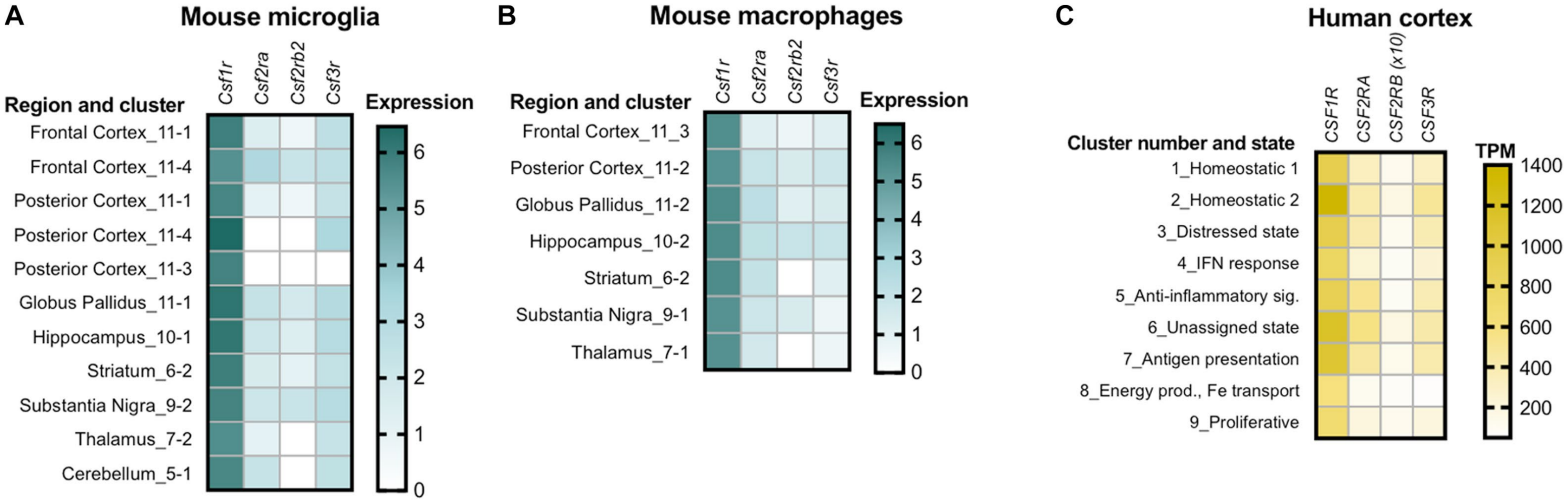
Variations in the expression of colony stimulating factor receptors among different populations of microglia and brain macrophages. **(A)** Variation among subclusters of microglia isolated from different regions of the adult mouse brain. Note the absence of transcripts for GM-CSFR and G-CSFR alone or in combination, in clusters of microglia isolated from the posterior cortex and the absence of transcripts for the βc high affinity subunit of GM-CSFR in thalamic and cerebellar microglia. **(B)** Mouse macrophages isolated from the same regions also show variable expression. Panels A and B were generated using data from dropviz.org (Saunders et al., 2018). **(C)** Heterogeneity of expression in human microglia isolated from the temporal and dorsolateral prefrontal cortex. The heat map was generated using data from Olah et al. (2018). To ensure visibility, the TPM values for *CSF2RB* were multiplied by a factor of 10. TPM, transcripts per million.

**Figure 3:**
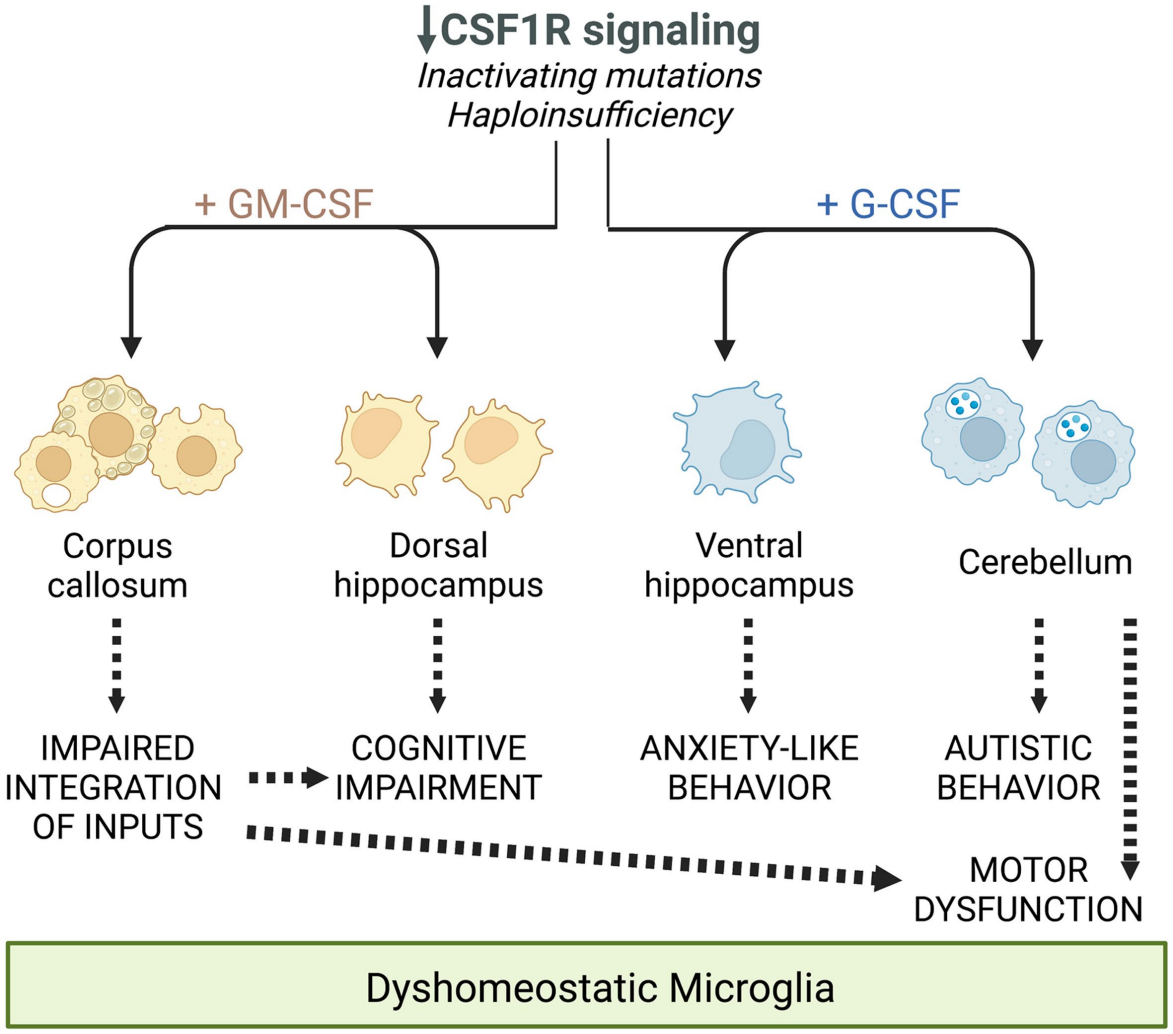
Schematic summarizing the differential and overlapping contributions of the increased expression of GM-CSF and G-CSF to the deficits observed in CRL mice, mediated through their regulation of microglia. Reprinted from Biundo et al. (2023b) with permission.

*Csf1r^+/−^* mice exhibit a slight early increase in microglial densities (Chitu et al., 2015; Arreola et al., 2021). This increase is established during development (Arreola et al., 2021) and, unless accompanied by other features such as morphological alterations or cell clustering, may not reflect a disease-related, reactive state. Consistent with the documented mitogenic effects of GM-CSF in microglia, we found that monoallelic deletion of *Csf2* normalized microglia densities in most brain regions of *Csf1r^+/−^* mice, with the exception of the cerebellum (Chitu et al., 2020). Furthermore, the demonstration that monoallelic deletion of *Csf2rb* in microglia also prevented the increase in forebrain microglial densities, provided formal evidence that the GM-CSF-induced microgliosis is due to direct signaling in microglia (Chitu et al., 2020). In contrast, monoallelic targeting of *Csf3* in *Csf1r^+/−^* mice had limited effects on microglia densities, normalizing their levels only in the corpus callosum, cerebellum and ventral hippocampus (Biundo et al., 2023b). This effect is likely indirect since G-CSF does not promote microglia proliferation (Giulian and Ingeman, 1988). Furthermore, the effects of G-CSF on microglia densities and morphology were at times dissociated, e.g., *Csf3* heterozygosity normalized the densities but not morphological alterations of microglia in the corpus callosum while having the reverse effect in the cortex of *Csf1r^+/−^* mice (Biundo et al., 2023b). The factors contributing to the apparently differential effects of GM-CSF and G-CSF in different populations of brain microglia remain to be elucidated. A possible explanation resides in the differential bioavailability of ligands in the mouse brain. For example, G-CSF is selectively elevated with age in the ventral, but not dorsal hippocampus (Porcher et al., 2021), a finding that could explain the dorsal region-specific response of hippocampal microglia to the monoallelic targeting of *Csf3* (Biundo et al., 2023b). Another explanation is suggested by single cell transcriptomic studies which reveal the heterogeneous expression of *Csf1r, Csf2ra, Csf2rb* and *Csf3r* among mouse and human microglia (Figure 2). Irrespective of their contribution to the elevation of microglia densities and their morphological alteration, gene expression studies provide additional evidence that both GM-CSF and G-CSF promote dyshomeostatic states in *Csf1r^+/−^* microglia. Transcriptomic profiling of microglia isolated from *Csf1r^+/−^* mice suggested a maladaptive phenotype which was significantly attenuated following monoallelic inactivation of *Csf2*. The expression of gene products that mediate synapse removal, trigger cellular senescence, neurotoxicity and oxidative stress was restored with consequent reduction of the histological evidence of oxidative damage and active demyelination/remyelination (Chitu et al., 2020). Similarly, analysis of cerebellar tissue from *Csf1r^+/−^; Csf3^+/−^* mice revealed that monoallelic targeting of *Csf3* reduced the production of C1q and its deposition on glutamatergic synapses and their consequent excessive removal by microglia (Biundo et al., 2023b). Overall, these studies show that under conditions in which the homeostatic signals provided by CSF-1R in microglia are attenuated, GM-CSF and G-CSF disrupt the normal functioning of microglia and brain homeostasis. The largely non-overlapping roles of GM-CSF and G-CSF in the development of CRL-like disease are summarized in Figure 3.

#### 2.2.7. Prophylactic potential of GM-CSF targeting in CRL

Accumulation of evidence that CRL is a primary microgliopathy has sparked an interest in evaluating the therapeutic potential of agents known to suppress microglia activation. Recently, a one-month course of minocycline, a suppressor of the inflammatory activation of microglia, was reported to partially rescue the cognitive dysfunction in 8-month-old *Csf1r^+/−^* male mice, to reduce myelin phagocytosis by *Csf1r^+/−^* microglia to wt levels and to contribute to the preservation of callosal myelin structure and of synaptic densities in the hippocampus (Li et al., 2023). It is unclear if the treatment would produce the same results in *Csf1r^+/−^* female mice, who were unaffected at the same age and have not been evaluated or treated at a later time point.

**Figure 4:**
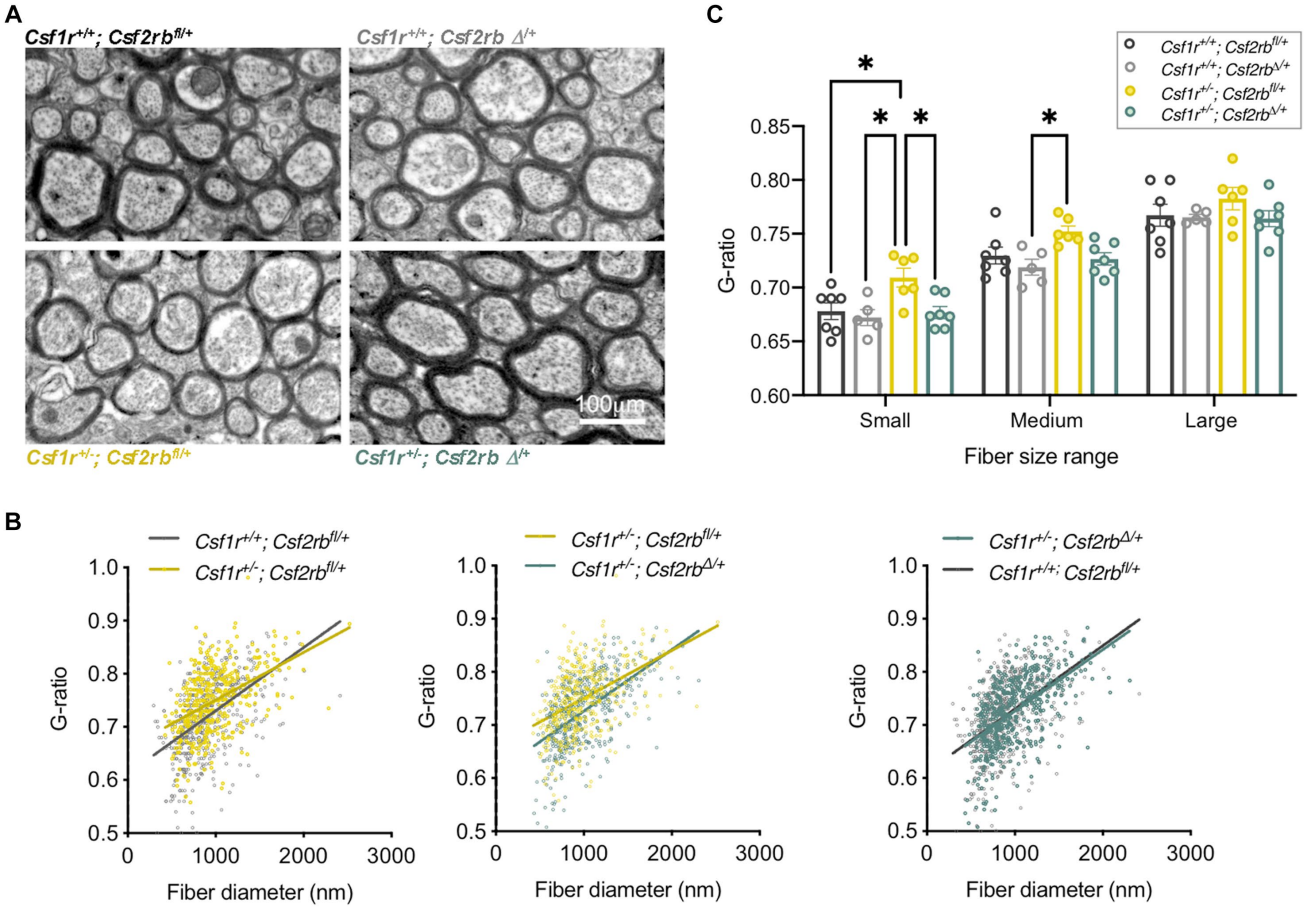
Inhibition of GM-CSF signaling in presymptomatic adult *Csf1r^+/−^* mice prevents demyelination. Two-month-old *Rosa26^creERT2/+^; Csf1r^+/+^; Csf2rb^fl/+^* and *Rosa26^creERT2/+^; Csf1r^+/−^; Csf2rb^fl/+^* mice (Chitu et al., 2020) were injected with Tamoxifen (2.5 mg, I.P.) to induce the monoallelic deletion of *Csf2rb* (*Csf2rb^Δ/+^*), or with vehicle (corn oil). Mice were euthanized and processed for electron microscopy at 9-months of age. **(A)** Myelin ultrastructure in callosal cross-sections. **(B)** *Csf1r^+/−^* mice exhibit an increase in the ratio of the axon versus myelinated fiber (G-ratio) in low and medium diameter axons (left panel *Csf1r^+/−^; Csf2rb^fl/+^*). This reflects the limited remyelination of the fibers following the loss of myelin. This phenotype is attenuated by removal of one *Csf2rb* allele (middle panel, *Csf1r^+/−^; Csf2rb^Δ/+^*) with *Csf1r^+/−^; Csf2rb^Δ/+^* becoming almost indistinguishable from wt (right panel). Individual G-ratios obtained from 5 to 7 mice/genotype are plotted against the fiber diameter. **(C)** Average G-ratios per mouse in the indicated axonal diameter ranges. Each dot on the chart represents average values from one mouse obtained as described previously (Chitu et al., 2020; Biundo et al., 2023b). Two-way ANOVA followed by Tukey’s multiple comparison test; *p < 0.05.

Clinical studies suggested that pre-symptomatic immunosuppression with glucocorticoids is protective in patients carrying pathogenic *CSF1R* variants associated with CRL (Tipton et al., 2021; Ali et al., 2022; Dulski et al., 2023). Further investigation in the mouse model showed that chronic prednisone administration initiated pre-symptomatically prevents the development of memory, motor coordination and social interaction deficits, as well as the associated demyelination, and neurodegeneration. Proteomic profiling *ex vivo* showed that prednisone administration suppressed a series of biological processes relevant to microglial activation and oligodendrocyte senescence (Chitu et al., 2023). These data suggest that CRL mutation carriers might benefit from prophylactic glucocorticoid treatment. The lack of improvement following steroid treatment in patients after the onset of demyelination (Konno et al., 2017a) suggests that prednisone usage might be restricted to the prophylaxis of CRL. In addition, chronic glucocorticoid administration can have significant side effects both peripherally (e.g., infection, diabetes, osteoporosis) and centrally (e.g., psychosis, depression, memory decline, seizures) (Witt and Sandoval, 2014). Thus, the exploration of downstream targets might provide therapeutic approaches with reduced side effects. Notably, glucocorticoids are known to suppress microglial activation by GM-CSF (Ganter et al., 1992; Tanaka et al., 1997) and suppression of GM-CSF signaling through monallelic targeting of *Csf2* in the mouse model of CRL prevented the development of cognitive, motor and olfactory deficits and the loss of myelin (Chitu et al., 2020). Importantly, inhibition of GM-CSF signaling in pre-symptomatic adult *Csf1r^+/−^* mice also prevents the loss of myelin (Figure 4), indicating that GM-CSF might also be a prophylactic target in CRL. Overall, these studies indicate that CRL may be managed by using pharmaceutical approaches.

## 3. Conclusion

**Figure 5:**
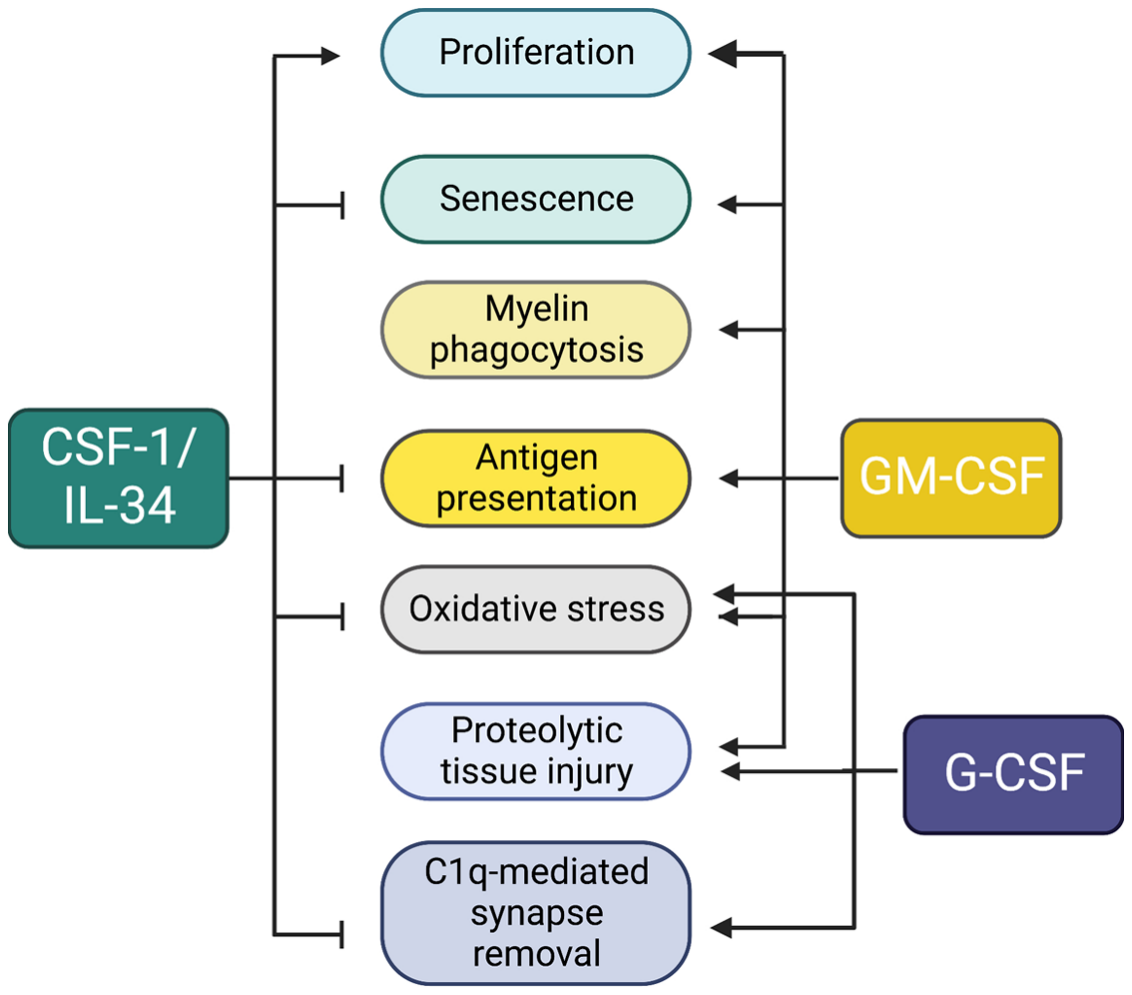
Unique and overlapping effects of colony stimulating factors in microglia. The diagram summarizes data obtained from studies *in vitro* (described in sections 1.2–1.4) and in *Csf1r^+/−^* mice (section 2.2.6).

Colony-stimulating factors regulate microglial development, maintenance and function in a non-overlapping and sometimes antagonistic fashion. A summary of the outcomes of their actions is presented in Figure 5. While CSF-1R ligands are constitutively expressed in the brain, transcripts for GM-CSF and G-CSF are barely detectable, but can be rapidly induced in response to infection or injury. Consistent with the more elevated expression of its ligands in normal brain, CSF-1R is the major mediator of microglial proliferation and is required for all microglial development and maintenance (Ginhoux et al., 2010; Huang et al., 2018). In contrast, neither GM-CSF nor G-CSF are required for microglial development and survival. GM-CSF is also a microglial mitogen (Schermer and Humpel, 2002; Xiao et al., 2002). However, in contrast to CSF-1, GM-CSF is proinflammatory and it promotes a demyelinating phenotype (Smith, 1993). G-CSF is not a microglial mitogen (Giulian and Ingeman, 1988), but is also proinflammatory, inducing a pro-oxidant phenotype in microglia (Basso et al., 2017). Studies in *Csf1r^+/−^* mice suggest that normal CNS homeostasis requires balanced signaling through all three microglial receptors (Chitu et al., 2020; Biundo et al., 2023b). CSF-1R signaling, in combination with low levels of GM-CSF and G-CSF, contribute to microglial homeostasis. In contrast, on the background of reduced CSF-1R signaling, elevation of either GM-CSF or G-CSF perturbs microglial homeostasis. The resulting altered states of microglia function might not be specific to CRL. Increased GM-CSF (Kostic et al., 2018) and G-CSF (Rumble et al., 2015; McGinley et al., 2020) levels and decreased microglial *Csf1r* (Werner et al., 2002) expression have also been reported in multiple sclerosis. Furthermore, increased GM-CSF levels were also reported in Alzheimer’s disease (Tarkowski et al., 2001). These findings suggest that imbalanced colony stimulating factor signaling might contribute to the pathogenesis of other neurodegenerative conditions.

## Author contributions

ES: Conceptualization, Formal analysis, Funding acquisition, Project administration, Resources, Supervision, Visualization, Writing – original draft, Writing – review & editing. FB: Data curation, Formal analysis, Investigation, Methodology, Writing – review & editing. SG: Investigation, Methodology, Writing – review & editing. VC: Formal analysis, Investigation, Methodology, Writing – review & editing.

## Footnotes

^1^https://singlecell.broadinstitute.org/single_cell/study/SCP263/aging-mouse-brain

## References

[ref1] Ali S. Tipton P. W. Koga S. Middlebrooks E. H. Josephs K. A. Strongosky A.. (2022). A novel Csf1R variant in a South Dakota family with Csf1R-related leukoencephalopathy. Parkinsonism Relat. Disord. 102, 51–53. doi: 10.1016/j.parkreldis.2022.07.016, PMID: 35940158

[ref2] Ali Z. S. Van Der Voorn J. P. Powers J. M. (2007). A comparative morphologic analysis of adult onset leukodystrophy with neuroaxonal spheroids and pigmented glia--a role for oxidative damage. J. Neuropathol. Exp. Neurol. 66, 660–672. doi: 10.1097/nen.0b013e3180986247, PMID: 17620991

[ref3] Aloisi F. Ria F. Adorini L. (2000). Regulation of T-cell responses by Cns antigen-presenting cells: different roles for microglia and astrocytes. Immunol. Today 21, 141–147. doi: 10.1016/S0167-5699(99)01512-1, PMID: 10689302

[ref4] Anderson S. R. Roberts J. M. Ghena N. Irvin E. A. Schwakopf J. Cooperstein I. B.. (2022). Neuronal apoptosis drives remodeling states of microglia and shifts in survival pathway dependence. elife 11:e76564. doi: 10.7554/eLife.76564, PMID: 35481836 PMC9071266

[ref5] Arreola M. A. Soni N. Crapser J. D. Hohsfield L. A. Elmore M. R. P. Matheos D. P.. (2021). Microglial dyshomeostasis drives perineuronal net and synaptic loss in a Csf1R(+/−) mouse model of Alsp, which can be rescued via Csf1R inhibitors. Sci. Adv. 7:eabg1601. doi: 10.1126/sciadv.abg1601.Print2021Aug 34433559 PMC8386924

[ref6] Badimon A. Strasburger H. J. Ayata P. Chen X. Nair A. Ikegami A.. (2020). Negative feedback control of neuronal activity by microglia. Nature 586, 417–423. doi: 10.1038/s41586-020-2777-8, PMID: 32999463 PMC7577179

[ref7] Baldwin G. C. Benveniste E. N. Chung G. Y. Gasson J. C. Golde D. W. (1993). Identification and characterization of a high-affinity granulocyte-macrophage colony-stimulating factor receptor on primary rat oligodendrocytes. Blood 82, 3279–3282. doi: 10.1182/blood.V82.11.3279.3279, PMID: 8241500

[ref8] Bartocci A. Mastrogiannis D. S. Migliorati G. Stockert R. J. Wolkoff A. W. Stanley E. R. (1987). Macrophages specifically regulate the concentration of their own growth factor in the circulation. Proc. Natl. Acad. Sci. U. S. A. 84, 6179–6183. doi: 10.1073/pnas.84.17.6179, PMID: 2819867 PMC299033

[ref9] Basso L. Lapointe T. K. Iftinca M. Marsters C. Hollenberg M. D. Kurrasch D. M.. (2017). Granulocyte-colony-stimulating factor (G-Csf) signaling in spinal microglia drives visceral sensitization following colitis. Proc. Natl. Acad. Sci. U. S. A. 114, 11235–11240. doi: 10.1073/pnas.1706053114, PMID: 28973941 PMC5651747

[ref10] Biundo F. Chitu V. Gökhan Ş. Chen E. Oppong-Asare J. Stanley E. R. (2023a). Trem2 enhances demyelination in the Csf1r+/− mouse model of leukoencephalopathy. Biomedicines in press 10.3390/biomedicines11082094 PMC10452898 37626591

[ref11] Biundo F. Chitu V. Shlager G. G. L. Park E. S. Gulinello M. E. Saha K.. (2021). Microglial reduction of colony stimulating factor-1 receptor expression is sufficient to confer adult onset leukodystrophy. Glia 69, 779–791. doi: 10.1002/glia.23929, PMID: 33079443 PMC8575656

[ref12] Biundo F. Chitu V. Tindi J. Burghardt N. S. Shlager G. G. L. Ketchum H. C.. (2023b). Elevated granulocyte colony stimulating factor (Csf) causes cerebellar deficits and anxiety in a model of Csf-1 receptor related leukodystrophy. Glia 71, 775–794. doi: 10.1002/glia.24310, PMID: 36433736 PMC9868112

[ref13] Block M. L. Zecca L. Hong J. S. (2007). Microglia-mediated neurotoxicity: uncovering the molecular mechanisms. Nat. Rev. Neurosci. 8, 57–69. doi: 10.1038/nrn2038, PMID: 17180163

[ref14] Bonvegna S. Straccia G. Golfre Andreasi N. Elia A. E. Marucci G. Di Bella D.. (2020). Parkinsonism and nigrostriatal damage secondary to Csf1R-related primary Microgliopathy. Mov. Disord. 35, 2360–2362. doi: 10.1002/mds.28290, PMID: 33009834

[ref15] Brown G. C. Neher J. J. (2014). Microglial phagocytosis of live neurons. Nat. Rev. Neurosci. 15, 209–216. doi: 10.1038/nrn3710, PMID: 24646669

[ref16] Bruttger J. Karram K. Wortge S. Regen T. Marini F. Hoppmann N.. (2015). Genetic cell ablation reveals clusters of local self-renewing microglia in the mammalian central nervous system. Immunity 43, 92–106. doi: 10.1016/j.immuni.2015.06.012, PMID: 26163371

[ref17] Caescu C. I. Guo X. Tesfa L. Bhagat T. D. Verma A. Zheng D.. (2015). Colony stimulating factor-1 receptor signaling networks inhibit mouse macrophage inflammatory responses by induction of microrna-21. Blood 125, e1–e13. doi: 10.1182/blood-2014-10-608000, PMID: 25573988 PMC4335087

[ref18] Cecchini M. G. Dominguez M. G. Mocci S. Wetterwald A. Felix R. Fleisch H.. (1994). Role of colony stimulating factor-1 in the establishment and regulation of tissue macrophages during postnatal development of the mouse. Development 120, 1357–1372. doi: 10.1242/dev.120.6.1357, PMID: 8050349

[ref19] Chitu V. Biundo F. Oppong-Asare J. Gokhan S. Aguilan J. T. Dulski J.. (2023). Prophylactic effect of chronic immunosuppression in a mouse model of Csf-1 receptor-related leukoencephalopathy. Glia. 71, 2664–2678. doi: 10.1002/glia.24446, PMID: 37519044 PMC10529087

[ref20] Chitu V. Biundo F. Shlager G. G. L. Park E. S. Wang P. Gulinello M. E.. (2020). Microglial homeostasis requires balanced Csf-1/Csf-2 receptor signaling. Cell Rep. 30, 3004–3019.e5. doi: 10.1016/j.celrep.2020.02.028 32130903 PMC7370656

[ref21] Chitu V. Biundo F. Stanley E. R. (2021). Colony stimulating factors in the nervous system. Semin. Immunol. 54:101511. doi: 10.1016/j.smim.2021.101511 34743926 PMC8671346

[ref22] Chitu V. Gokhan S. Gulinello M. Branch C. A. Patil M. Basu R.. (2015). Phenotypic characterization of a Csf1r haploinsufficient mouse model of adult-onset leukodystrophy with axonal spheroids and pigmented glia (Alsp). Neurobiol. Dis. 74, 219–228. doi: 10.1016/j.nbd.2014.12.001, PMID: 25497733 PMC4323933

[ref23] Chitu V. Gokhan S. Stanley E. R. (2022). Modeling Csf-1 receptor deficiency diseases - how close are we? FEBS J. 289, 5049–5073. doi: 10.1111/febs.16085, PMID: 34145972 PMC8684558

[ref24] Chitu V. Stanley E. R. (2006). Colony-stimulating factor-1 in immunity and inflammation. Curr. Opin. Immunol. 18, 39–48. doi: 10.1016/j.coi.2005.11.006, PMID: 16337366

[ref25] Clare A. J. Day R. C. Empson R. M. Hughes S. M. (2018). Transcriptome profiling of layer 5 Intratelencephalic projection neurons from the mature mouse motor cortex. Front. Mol. Neurosci. 11:410. doi: 10.3389/fnmol.2018.00410, PMID: 30483051 PMC6240696

[ref26] Davalos D. Grutzendler J. Yang G. Kim J. V. Zuo Y. Jung S.. (2005). Atp mediates rapid microglial response to local brain injury in vivo. Nat. Neurosci. 8, 752–758. doi: 10.1038/nn1472, PMID: 15895084

[ref27] David S. Kroner A. (2011). Repertoire of microglial and macrophage responses after spinal cord injury. Nat. Rev. Neurosci. 12, 388–399. doi: 10.1038/nrn3053, PMID: 21673720

[ref28] Delaney C. Farrell M. Doherty C. P. Brennan K. O'keeffe E. Greene C.. (2020). Attenuated Csf-1R signalling drives cerebrovascular pathology. EMBO Mol. Med. e12889. doi: 10.15252/emmm.202012889 33350588 PMC7863388

[ref29] Diederich K. Sevimli S. Dorr H. Kosters E. Hoppen M. Lewejohann L.. (2009). The role of granulocyte-colony stimulating factor (G-Csf) in the healthy brain: a characterization of G-Csf-deficient mice. J. Neurosci. 29, 11572–11581. doi: 10.1523/JNEUROSCI.0453-09.2009, PMID: 19759304 PMC6665757

[ref30] Dikmen H. O. Hemmerich M. Lewen A. Hollnagel J. O. Chausse B. Kann O. (2020). Gm-Csf induces noninflammatory proliferation of microglia and disturbs electrical neuronal network rhythms in situ. J. Neuroinflammation 17:235. doi: 10.1186/s12974-020-01903-4, PMID: 32782006 PMC7418331

[ref31] Ding D. X.-H. Rivas C. I. Heaney M. L. Raines M. A. Vera J. C. Golde D. W. (1994). The a subunit of the human granulocyte-macrophage colony-stimulating factor receptor signals for glucose transport via a phosphorylation-independent pathway. Proc. Natl. Acad. Sci. U. S. A. 91, 2537–2541. doi: 10.1073/pnas.91.7.2537, PMID: 8146150 PMC43404

[ref32] Dougan M. Dranoff G. Dougan S. K. (2019). Gm-Csf, Il-3, and Il-5 family of cytokines: regulators of inflammation. Immunity 50, 796–811. doi: 10.1016/j.immuni.2019.03.022, PMID: 30995500 PMC12512237

[ref33] Dulski J. Heckman M. G. Nowak J. Wszolek Z. (2023). Protective effect of glucocorticoids against symptomatic disease in Csf1R variant carriers. Move. Disord. In Press 10.1002/mds.29504 37309919

[ref34] Easley-Neal C. Foreman O. Sharma N. Zarrin A. A. Weimer R. M. (2019). Csf1R ligands Il-34 and Csf1 are differentially required for microglia development and maintenance in White and gray matter brain regions. Front. Immunol. 10:2199. doi: 10.3389/fimmu.2019.02199, PMID: 31616414 PMC6764286

[ref35] Elmore M. R. Najafi A. R. Koike M. A. Dagher N. N. Spangenberg E. E. Rice R. A.. (2014). Colony-stimulating factor 1 receptor signaling is necessary for microglia viability, unmasking a microglia progenitor cell in the adult brain. Neuron 82, 380–397. doi: 10.1016/j.neuron.2014.02.040, PMID: 24742461 PMC4161285

[ref36] Farzam-Kia N. Lemaitre F. Carmena Moratalla A. Carpentier Solorio Y. Da Cal S. Jamann H.. (2023). Granulocyte-macrophage colony-stimulating factor-stimulated human macrophages demonstrate enhanced functions contributing to T-cell activation. Immunol. Cell Biol. 101, 65–77. doi: 10.1111/imcb.12600, PMID: 36260372

[ref37] Fernandez-Arjona M. D. M. Grondona J. M. Granados-Duran P. Fernandez-Llebrez P. Lopez-Avalos M. D. (2017). Microglia morphological categorization in a rat model of Neuroinflammation by hierarchical cluster and principal components analysis. Front. Cell. Neurosci. 11:235. doi: 10.3389/fncel.2017.00235, PMID: 28848398 PMC5550745

[ref38] Frydecka D. Krzystek-Korpacka M. Lubeiro A. Stramecki F. Stanczykiewicz B. Beszlej J. A.. (2018). Profiling inflammatory signatures of schizophrenia: a cross-sectional and meta-analysis study. Brain Behav. Immun. 71, 28–36. doi: 10.1016/j.bbi.2018.05.002, PMID: 29730395

[ref39] Fu R. Shen Q. Xu P. Luo J. J. Tang Y. (2014). Phagocytosis of microglia in the central nervous system diseases. Mol. Neurobiol. 49, 1422–1434. doi: 10.1007/s12035-013-8620-6, PMID: 24395130 PMC4012154

[ref40] Ganter S. Northoff H. Mannel D. Gebicke-Harter P. J. (1992). Growth control of cultured microglia. J. Neurosci. Res. 33, 218–230. doi: 10.1002/jnr.490330205, PMID: 1333539

[ref41] Ginhoux F. Greter M. Leboeuf M. Nandi S. See P. Gokhan S.. (2010). Fate mapping analysis reveals that adult microglia derive from primitive macrophages. Science 330, 841–845. doi: 10.1126/science.1194637, PMID: 20966214 PMC3719181

[ref42] Ginhoux F. Prinz M. (2015). Origin of microglia: current concepts and past controversies. Cold Spring Harb. Perspect. Biol. 7. doi: 10.1101/cshperspect.a020537 PMC4526747 26134003

[ref43] Giulian D. Ingeman J. E. (1988). Colony-stimulating factors as promoters of ameboid microglia. J. Neurosci. 8, 4707–4717. doi: 10.1523/JNEUROSCI.08-12-04707.1988, PMID: 3058881 PMC6569554

[ref44] Goldmann T. Wieghofer P. Jordao M. J. Prutek F. Hagemeyer N. Frenzel K.. (2016). Origin, fate and dynamics of macrophages at central nervous system interfaces. Nat. Immunol. 17, 797–805. doi: 10.1038/ni.3423, PMID: 27135602 PMC4968048

[ref45] Gomez Perdiguero E. Klapproth K. Schulz C. Busch K. Azzoni E. Crozet L.. (2015). Tissue-resident macrophages originate from yolk-sac-derived erythro-myeloid progenitors. Nature 518, 547–551. doi: 10.1038/nature13989, PMID: 25470051 PMC5997177

[ref46] Greter M. Lelios I. Pelczar P. Hoeffel G. Price J. Leboeuf M.. (2012). Stroma-derived interleukin-34 controls the development and maintenance of langerhans cells and the maintenance of microglia. Immunity 37, 1050–1060. doi: 10.1016/j.immuni.2012.11.001, PMID: 23177320 PMC4291117

[ref47] Guerreiro R. Kara E. Le Ber I. Bras J. Rohrer J. D. Taipa R.. (2013). Genetic analysis of inherited leukodystrophies: genotype-phenotype correlations in the Csf1R gene. JAMA Neurol. 70, 875–882. doi: 10.1001/jamaneurol.2013.698, PMID: 23649896 PMC4204151

[ref48] Hagemeyer N. Kierdorf K. Frenzel K. Xue J. Ringelhan M. Abdullah Z.. (2016). Transcriptome-based profiling of yolk sac-derived macrophages reveals a role for Irf8 in macrophage maturation. EMBO J. 35, 1730–1744. doi: 10.15252/embj.201693801, PMID: 27412700 PMC5010043

[ref49] Hamilton J. A. (2020). Gm-Csf in inflammation. J. Exp. Med. 217:e20190945. doi: 10.1084/jem.20190945, PMID: 31611249 PMC7037240

[ref50] Hammond T. R. Dufort C. Dissing-Olesen L. Giera S. Young A. Wysoker A.. (2019). Single-cell Rna sequencing of microglia throughout the mouse lifespan and in the injured brain reveals complex cell-state changes. Immunity 50:e6, 253–271.e6. doi: 10.1016/j.immuni.2018.11.004 PMC6655561 30471926

[ref51] Hanisch U. K. Kettenmann H. (2007). Microglia: active sensor and versatile effector cells in the normal and pathologic brain. Nat. Neurosci. 10, 1387–1394. doi: 10.1038/nn1997, PMID: 17965659

[ref52] Hansmann F. Herder V. Kalkuhl A. Haist V. Zhang N. Schaudien D.. (2012). Matrix metalloproteinase-12 deficiency ameliorates the clinical course and demyelination in Theiler's murine encephalomyelitis. Acta Neuropathol. 124, 127–142. doi: 10.1007/s00401-012-0942-3, PMID: 22271152

[ref53] Haves-Zburof D. Paperna T. Gour-Lavie A. Mandel I. Glass-Marmor L. Miller A. (2011). Cathepsins and their endogenous inhibitors cystatins: expression and modulation in multiple sclerosis. J. Cell. Mol. Med. 15, 2421–2429. doi: 10.1111/j.1582-4934.2010.01229.x, PMID: 21143385 PMC3822953

[ref54] Hiyoshi M. Hashimoto M. Yukihara M. Bhuyan F. Suzu S. (2013). M-Csf receptor mutations in hereditary diffuse leukoencephalopathy with spheroids impair not only kinase activity but also surface expression. Biochem. Biophys. Res. Commun. 440, 589–593. doi: 10.1016/j.bbrc.2013.09.141, PMID: 24120500

[ref55] Hoeffel G. Wang Y. Greter M. See P. Teo P. Malleret B.. (2012). Adult Langerhans cells derive predominantly from embryonic fetal liver monocytes with a minor contribution of yolk sac-derived macrophages. J. Exp. Med. 209, 1167–1181. doi: 10.1084/jem.20120340, PMID: 22565823 PMC3371735

[ref56] Hohsfield L. A. Najafi A. R. Ghorbanian Y. Soni N. Crapser J. Figueroa Velez D. X.. (2021). Subventricular zone/white matter microglia reconstitute the empty adult microglial niche in a dynamic wave. elife 10:e66738. doi: 10.7554/eLife.66738, PMID: 34423781 PMC8425950

[ref57] Hu X. Leak R. K. Shi Y. Suenaga J. Gao Y. Zheng P.. (2015). Microglial and macrophage polarization-new prospects for brain repair. Nat. Rev. Neurol. 11, 56–64. doi: 10.1038/nrneurol.2014.207, PMID: 25385337 PMC4395497

[ref58] Huang Y. Xu Z. Xiong S. Sun F. Qin G. Hu G.. (2018). Repopulated microglia are solely derived from the proliferation of residual microglia after acute depletion. Nat. Neurosci. 21, 530–540. doi: 10.1038/s41593-018-0090-8, PMID: 29472620

[ref59] Hume D. A. Caruso M. Ferrari-Cestari M. Summers K. M. Pridans C. Irvine K. M. (2020). Phenotypic impacts of Csf1R deficiencies in humans and model organisms. J. Leukoc. Biol. 107, 205–219. doi: 10.1002/JLB.MR0519-143R, PMID: 31330095

[ref60] Ishiguro A. Inoue K. Nakahata T. Nishihira H. Kojima S. Ueda K.. (1996). Reference intervals for serum granulocyte colony-stimulating factor levels in children. J. Pediatr. 128, 208–212. doi: 10.1016/S0022-3476(96)70391-8, PMID: 8636813

[ref61] Ivy G. O. Killackey H. P. (1981). The ontogeny of the distribution of callosal projection neurons in the rat parietal cortex. J. Comp. Neurol. 195, 367–389. doi: 10.1002/cne.901950302, PMID: 6162864

[ref62] Janowska-Wieczorek A. Belch A. R. Jacobs A. Bowen D. Padua R. A. Paietta E.. (1991). Increased circulating colony-stimulating factor-1 in patients with preleukemia, leukemia, and lymphoid malignancies. Blood 77, 1796–1803. doi: 10.1182/blood.V77.8.1796.1796, PMID: 2015402

[ref63] Kadar E. Sureda A. Mangues M. A. Ingles-Esteve J. Valls A. Garcia J. (1997). Serum levels of G-Csf, Il-3, Il-6 and gm-Csf after a single intraperitoneal dose of rhG-Csf in lethally irradiated B6D2F1 mice. Acta Haematol. 98, 119–124. doi: 10.1159/000203604 9352740

[ref64] Kana V. Desland F. A. Casanova-Acebes M. Ayata P. Badimon A. Nabel E.. (2019). Csf-1 controls cerebellar microglia and is required for motor function and social interaction. J. Exp. Med. 216, 2265–2281. doi: 10.1084/jem.20182037, PMID: 31350310 PMC6781012

[ref65] Karle K. N. Biskup S. Schule R. Schweitzer K. J. Kruger R. Bauer P.. (2013). De novo mutations in hereditary diffuse leukoencephalopathy with axonal spheroids (Hdls). Neurology 81, 2039–2044. doi: 10.1212/01.wnl.0000436945.01023.ac, PMID: 24198292

[ref66] Kawakami I. Iseki E. Kasanuki K. Minegishi M. Sato K. Hino H.. (2016). A family with hereditary diffuse leukoencephalopathy with spheroids caused by a novel c.2442+2T>C mutation in the Csf1R gene. J. Neurol. Sci. 367, 349–355. doi: 10.1016/j.jns.2016.06.013, PMID: 27423618

[ref67] Kempthorne L. Yoon H. Madore C. Smith S. Wszolek Z. K. Rademakers R.. (2020). Loss of homeostatic microglial phenotype in Csf1R-related leukoencephalopathy. Acta Neuropathol. Commun. 8:72. doi: 10.1186/s40478-020-00947-0, PMID: 32430064 PMC7236286

[ref68] Kierdorf K. Erny D. Goldmann T. Sander V. Schulz C. Perdiguero E. G.. (2013). Microglia emerge from erythromyeloid precursors via Pu.1- and Irf8-dependent pathways. Nat. Neurosci. 16, 273–280. doi: 10.1038/nn.3318, PMID: 23334579

[ref69] Kim S. I. Jeon B. Bae J. Won J. K. Kim H. J. Yim J.. (2019). An autopsy proven case of Csf1R-mutant adult-onset leukoencephalopathy with axonal spheroids and pigmented glia (Alsp) with premature ovarian failure. Exp. Neurobiol. 28, 119–129. doi: 10.5607/en.2019.28.1.119, PMID: 30853829 PMC6401550

[ref70] Kim E. J. Shin J. H. Lee J. H. Kim J. H. Na D. L. Suh Y. L.. (2015). Adult-onset leukoencephalopathy with axonal spheroids and pigmented glia linked Csf1R mutation: report of four Korean cases. J. Neurol. Sci. 349, 232–238. doi: 10.1016/j.jns.2014.12.021, PMID: 25563800

[ref71] Kiriyama R. Chichibu K. Matsuno T. Ohsawa N. (1993). Sensitive chemiluminescent immunoassay for human granulocyte colony-stimulating factor (G-Csf) in clinical applications. Clin. Chim. Acta 220, 201–209. doi: 10.1016/0009-8981(93)90048-9, PMID: 7509266

[ref72] Konno T. Broderick D. F. Mezaki N. Isami A. Kaneda D. Tashiro Y.. (2017a). Diagnostic value of brain calcifications in adult-onset leukoencephalopathy with axonal spheroids and pigmented glia. AJNR Am. J. Neuroradiol. 38, 77–83. doi: 10.3174/ajnr.A4938, PMID: 27633805 PMC5233547

[ref73] Konno T. Kasanuki K. Ikeuchi T. Dickson D. W. Wszolek Z. K. (2018). Csf1R-related leukoencephalopathy: a major player in primary microgliopathies. Neurology 91, 1092–1104. doi: 10.1212/WNL.0000000000006642, PMID: 30429277 PMC6329328

[ref74] Konno T. Tada M. Tada M. Koyama A. Nozaki H. Harigaya Y.. (2014a). Haploinsufficiency of Csf-1R and clinicopathologic characterization in patients with Hdls. Neurology 82, 139–148. doi: 10.1212/WNL.0000000000000046, PMID: 24336230 PMC3937843

[ref75] Konno T. Tada M. Tada M. Nishizawa M. Ikeuchi T. (2014b). Hereditary diffuse leukoencephalopathy with spheroids (Hdls): a review of the literature on its clinical characteristics and mutations in the Colony-stimulating Factor-1 receptor gene. Brain Nerve 66, 581–590. doi: 10.11477/mf.1416101796 PMID: 24807373

[ref76] Konno T. Yoshida K. Mizuno T. Kawarai T. Tada M. Nozaki H.. (2017b). Clinical and genetic characterization of adult-onset leukoencephalopathy with axonal spheroids and pigmented glia associated with Csf1R mutation. Eur. J. Neurol. 24, 37–45. doi: 10.1111/ene.13125, PMID: 27680516 PMC5215554

[ref77] Kostic M. Zivkovic N. Cvetanovic A. Stojanovic I. (2018). Granulocyte-macrophage colony-stimulating factor as a mediator of autoimmunity in multiple sclerosis. J. Neuroimmunol. 323, 1–9. doi: 10.1016/j.jneuroim.2018.07.002, PMID: 30196820

[ref78] Kozareva V. Martin C. Osorno T. Rudolph S. Guo C. Vanderburg C.. (2021). A transcriptomic atlas of mouse cerebellar cortex comprehensively defines cell types. Nature 598, 214–219. doi: 10.1038/s41586-021-03220-z, PMID: 34616064 PMC8494635

[ref79] Kraya T. Quandt D. Pfirrmann T. Kindermann A. Lampe L. Schroeter M. L.. (2019). Functional characterization of a novel Csf1R mutation causing hereditary diffuse leukoencephalopathy with spheroids. Mol. Genet. Genom. Med. 7:e00595. doi: 10.1002/mgg3.595, PMID: 30729751 PMC6465730

[ref80] Krieger M. Both M. Kranig S. A. Pitzer C. Klugmann M. Vogt G.. (2012). The hematopoietic cytokine granulocyte-macrophage colony stimulating factor is important for cognitive functions. Sci. Rep. 2:697. doi: 10.1038/srep00697, PMID: 23019518 PMC3458247

[ref81] Laske C. Stellos K. Stransky E. Leyhe T. Gawaz M. (2009). Decreased plasma levels of granulocyte-colony stimulating factor (G-Csf) in patients with early Alzheimer's disease. J. Alzheimers Dis. 17, 115–123. doi: 10.3233/JAD-2009-1017, PMID: 19494436

[ref82] Lawson L. J. Perry V. H. Dri P. Gordon S. (1990). Heterogeneity in the distribution and morphology of microglia in the normal adult mouse brain. Neuroscience 19, 151–170. 10.1016/0306-4522(90)90229-w 2089275

[ref83] Lee S. C. Liu W. Brosnan C. F. Dickson D. W. (1994). Gm-Csf promotes proliferation of human fetal and adult microglia in primary cultures. Glia 12, 309–318. doi: 10.1002/glia.440120407, PMID: 7890333

[ref84] Lee S. C. Liu W. Roth P. Dickson D. W. Berman J. W. Brosnan C. F. (1993). Macrophage colony-stimulating factor in human fetal astrocytes and microglia. Differential regulation by cytokines and lipopolysaccharide, and modulation of class ii Mhc on microglia. J. Immunol. 150, 594–604. doi: 10.4049/jimmunol.150.2.594, PMID: 8419491

[ref85] Lelli A. Gervais A. Colin C. Cheret C. Ruiz De Almodovar C. Carmeliet P.. (2013). The Nadph oxidase Nox2 regulates Vegfr1/Csf-1R-mediated microglial chemotaxis and promotes early postnatal infiltration of phagocytes in the subventricular zone of the mouse cerebral cortex. Glia 61, 1542–1555. doi: 10.1002/glia.22540, PMID: 23836548

[ref86] Leng C. Lu L. Wang G. Zhang Y. Xu Y. Lin X.. (2019). A novel dominant-negative mutation of the Csf1R gene causes adult-onset leukoencephalopathy with axonal spheroids and pigmented glia. Am. J. Transl. Res. 11, 6093–6101. PMID: 31632577 PMC6789214

[ref87] Li X. Hu B. Guan X. Wang Z. Zhou Y. Sun H.. (2023). Minocycline protects against microgliopathy in a Csf1r haplo-insufficient mouse model of adult-onset leukoencephalopathy with axonal spheroids and pigmented glia (Alsp). J. Neuroinflammation 20:134. doi: 10.1186/s12974-023-02774-1, PMID: 37259140 PMC10234026

[ref88] Liuzzo J. P. Petanceska S. S. Moscatelli D. Devi L. A. (1999). Inflammatory mediators regulate cathepsin S in macrophages and microglia: a role in attenuating heparan sulfate interactions. Mol. Med. 5, 320–333. doi: 10.1007/BF03402068, PMID: 10390548 PMC2230418

[ref89] Lobsiger C. S. Boillee S. Pozniak C. Khan A. M. Mcalonis-Downes M. Lewcock J. W.. (2013). C1q induction and global complement pathway activation do not contribute to Als toxicity in mutant Sod1 mice. Proc. Natl. Acad. Sci. U. S. A. 110, E4385–E4392. doi: 10.1073/pnas.1318309110, PMID: 24170856 PMC3831990

[ref90] Luo J. Elwood F. Britschgi M. Villeda S. Zhang H. Ding Z.. (2013). Colony-stimulating factor 1 receptor (Csf1R) signaling in injured neurons facilitates protection and survival. J. Exp. Med. 210, 157–172. doi: 10.1084/jem.20120412, PMID: 23296467 PMC3549715

[ref91] Lynch D. S. Jaunmuktane Z. Sheerin U. M. Phadke R. Brandner S. Milonas I.. (2016). Hereditary leukoencephalopathy with axonal spheroids: a spectrum of phenotypes from Cns vasculitis to parkinsonism in an adult onset leukodystrophy series. J. Neurol. Neurosurg. Psychiatry 87, 512–519. doi: 10.1136/jnnp-2015-310788, PMID: 25935893 PMC4853550

[ref92] Lynch D. S. Rodrigues Brandão de Paiva A. Zhang W. J. Bugiardini E. Freua F. Tavares Lucato L.. (2017). Clinical and genetic characterization of leukoencephalopathies in adults. Brain 140, 1204–1211. doi: 10.1093/brain/awx045, PMID: 28334938 PMC5405235

[ref880] Magnus T. Korn T. Jung S. (2004). Chronically stimulated microglial cells do no longer alter their immune functions in response to the phagocytosis of apoptotic cells. J Neuroimmunol. 155, 64–72. doi: 10.1016/j.jneuroim.2004.06.002, PMID: 15342197

[ref93] Mateen F. J. Keegan B. M. Krecke K. Parisi J. E. Trenerry M. R. Pittock S. J. (2010). Sporadic leucodystrophy with neuroaxonal spheroids: persistence of Dwi changes and neurocognitive profiles: a case study. J. Neurol. Neurosurg. Psychiatry 81, 619–622. doi: 10.1136/jnnp.2008.169243, PMID: 20176606

[ref94] Matyszak M. K. Denis-Donini S. Citterio S. Longhi R. Granucci F. Ricciardi-Castagnoli P. (1999). Microglia induce myelin basic protein-specific T cell anergy or T cell activation, according to their state of activation. Eur. J. Immunol. 29, 3063–3076. doi: 10.1002/(SICI)1521-4141(199910)29:10<3063::AID-IMMU3063>3.0.CO;2-G, PMID: 10540317

[ref95] Mcginley A. M. Sutton C. E. Edwards S. C. Leane C. M. Decourcey J. Teijeiro A.. (2020). Interleukin-17A serves a priming role in autoimmunity by recruiting Il-1beta-producing myeloid cells that promote pathogenic T cells. Immunity 52:e6. doi: 10.1016/j.immuni.2020.01.002 32023490

[ref96] Mclay R. N. Kimura M. Banks W. A. Kastin A. J. (1997). Granulocyte-macrophage colony-stimulating factor crosses the blood--brain and blood--spinal cord barriers. Brain 120, 2083–2091. doi: 10.1093/brain/120.11.2083, PMID: 9397023

[ref97] Meyer-Ohlendorf M. Braczynski A. Al-Qaisi O. Gessler F. Biskup S. Weise L.. (2015). Comprehensive diagnostics in a case of hereditary diffuse leukodystrophy with spheroids. BMC Neurol. 15:103. doi: 10.1186/s12883-015-0368-3, PMID: 26141177 PMC4491230

[ref98] Miura T. Mezaki N. Konno T. Iwasaki A. Hara N. Miura M.. (2018). Identification and functional characterization of novel mutations including frameshift mutation in exon 4 of Csf1R in patients with adult-onset leukoencephalopathy with axonal spheroids and pigmented glia. J. Neurol. 265, 2415–2424. doi: 10.1007/s00415-018-9017-2, PMID: 30136118 PMC6182692

[ref99] Munro D. A. D. Bradford B. M. Mariani S. A. Hampton D. W. Vink C. S. Chandran S.. (2020). Cns macrophages differentially rely on an intronic Csf1r enhancer for their development. Development 147:dev194449. doi: 10.1242/dev.194449, PMID: 33323375 PMC7758622

[ref100] Nakajima K. Kohsaka S. (2004). Microglia: neuroprotective and neurotrophic cells in the central nervous system. Curr. Drug Targets Cardiovasc. Haematol. Disord. 4, 65–84. doi: 10.2174/1568006043481284 15032653

[ref101] Nandi S. Gokhan S. Dai X. M. Wei S. Enikolopov G. Lin H.. (2012). The Csf-1 receptor ligands Il-34 and Csf-1 exhibit distinct developmental brain expression patterns and regulate neural progenitor cell maintenance and maturation. Dev. Biol. 367, 100–113. doi: 10.1016/j.ydbio.2012.03.026, PMID: 22542597 PMC3388946

[ref102] Neumann H. Kotter M. R. Franklin R. J. (2009). Debris clearance by microglia: an essential link between degeneration and regeneration. Brain 132, 288–295. doi: 10.1093/brain/awn109, PMID: 18567623 PMC2640215

[ref103] Nicholson A. M. Baker M. C. Finch N. A. Rutherford N. J. Wider C. Graff-Radford N. R.. (2013). Csf1R mutations link Pold and Hdls as a single disease entity. Neurology 80, 1033–1040. doi: 10.1212/WNL.0b013e31828726a7, PMID: 23408870 PMC3653204

[ref104] Nimmerjahn A. Kirchhoff F. Helmchen F. (2005). Resting microglial cells are highly dynamic surveillants of brain parenchyma *in vivo*. Science 308, 1314–1318. doi: 10.1126/science.1110647, PMID: 15831717

[ref105] Olah M. Patrick E. Villani A. C. Xu J. White C. C. Ryan K. J.. (2018). A transcriptomic atlas of aged human microglia. Nat. Commun. 9:539. doi: 10.1038/s41467-018-02926-5, PMID: 29416036 PMC5803269

[ref106] Oyanagi K. Kinoshita M. Suzuki-Kouyama E. Inoue T. Nakahara A. Tokiwai M.. (2017). Adult onset leukoencephalopathy with axonal spheroids and pigmented glia (Alsp) and Nasu-Hakola disease: lesion staging and dynamic changes of axons and microglial subsets. Brain Pathol. 27, 748–769. doi: 10.1111/bpa.12443, PMID: 27608278 PMC8029200

[ref107] Paolicelli R. C. Bolasco G. Pagani F. Maggi L. Scianni M. Panzanelli P.. (2011). Synaptic pruning by microglia is necessary for normal brain development. Science 333, 1456–1458. doi: 10.1126/science.1202529, PMID: 21778362

[ref108] Parkhurst C. N. Yang G. Ninan I. Savas J. N. Yates J. R. Lafaille J. J.. (2013). Microglia promote learning-dependent synapse formation through brain-derived neurotrophic factor. Cells 155, 1596–1609. doi: 10.1016/j.cell.2013.11.030, PMID: 24360280 PMC4033691

[ref109] Porcher L. Bruckmeier S. Burbano S. D. Finnell J. E. Gorny N. Klett J.. (2021). Aging triggers an upregulation of a multitude of cytokines in the male and especially the female rodent hippocampus but more discrete changes in other brain regions. J. Neuroinflammation 18:219. doi: 10.1186/s12974-021-02252-6, PMID: 34551810 PMC8459490

[ref110] Prinz M. Erny D. Hagemeyer N. (2017). Ontogeny and homeostasis of Cns myeloid cells. Nat. Immunol. 18, 385–392. doi: 10.1038/ni.3703, PMID: 28323268

[ref111] Rademakers R. Baker M. Nicholson A. M. Rutherford N. J. Finch N. Soto-Ortolaza A.. (2011). Mutations in the colony stimulating factor 1 receptor (Csf1R) gene cause hereditary diffuse leukoencephalopathy with spheroids. Nat. Genet. 44, 200–205. doi: 10.1038/ng.1027, PMID: 22197934 PMC3267847

[ref112] Re F. Belyanskaya S. L. Riese R. J. Cipriani B. Fischer F. R. Granucci F.. (2002). Granulocyte-macrophage colony-stimulating factor induces an expression program in neonatal microglia that primes them for antigen presentation. J. Immunol. 169, 2264–2273. doi: 10.4049/jimmunol.169.5.2264, PMID: 12193691

[ref113] Riku Y. Ando T. Goto Y. Mano K. Iwasaki Y. Sobue G.. (2014). Early pathologic changes in hereditary diffuse leukoencephalopathy with spheroids. J. Neuropathol. Exp. Neurol. 73, 1183–1190. doi: 10.1097/NEN.0000000000000139, PMID: 25383640

[ref114] Roth L. Van Dam D. Van Der Donckt C. Schrijvers D. M. Lemmens K. Van Brussel I.. (2015). Impaired gait pattern as a sensitive tool to assess hypoxic brain damage in a novel mouse model of atherosclerotic plaque rupture. Physiol. Behav. 139, 397–402. doi: 10.1016/j.physbeh.2014.11.047, PMID: 25449385

[ref115] Rumble J. M. Huber A. K. Krishnamoorthy G. Srinivasan A. Giles D. A. Zhang X.. (2015). Neutrophil-related factors as biomarkers in Eae and Ms. J. Exp. Med. 212, 23–35. doi: 10.1084/jem.20141015, PMID: 25559893 PMC4291533

[ref116] Ryan G. R. Dai X. M. Dominguez M. G. Tong W. Chuan F. Chisholm O.. (2001). Rescue of the colony-stimulating factor 1 (Csf-1)-nullizygous mouse (Csf1(op)/Csf1(op)) phenotype with a Csf-1 transgene and identification of sites of local Csf-1 synthesis. Blood 98, 74–84. doi: 10.1182/blood.V98.1.74, PMID: 11418465

[ref117] Safaiyan S. Besson-Girard S. Kaya T. Cantuti-Castelvetri L. Liu L. Ji H.. (2021). White matter aging drives microglial diversity. Neuron 109:e10. doi: 10.1016/j.neuron.2021.01.027 33606969

[ref118] Sasaki A. Yokoo H. Naito M. Kaizu C. Shultz L. D. Nakazato Y. (2000). Effects of macrophage-colony-stimulating factor deficiency on the maturation of microglia and brain macrophages and on their expression of scavenger receptor. Neuropathology 20, 134–142. doi: 10.1046/j.1440-1789.2000.00286.x, PMID: 10935450

[ref119] Saunders A. Macosko E. Z. Wysoker A. Goldman M. Krienen F. M. De Rivera H.. (2018). Molecular diversity and specializations among the cells of the adult mouse brain. Cells 174:e16. doi: 10.1016/j.cell.2018.07.028 PMC6447408 30096299

[ref120] Schermer C. Humpel C. (2002). Granulocyte macrophage-colony stimulating factor activates microglia in rat cortex organotypic brain slices. Neurosci. Lett. 328, 180–184. doi: 10.1016/S0304-3940(02)00496-2, PMID: 12133583

[ref121] Schneider A. Kruger C. Steigleder T. Weber D. Pitzer C. Laage R.. (2005). The hematopoietic factor G-Csf is a neuronal ligand that counteracts programmed cell death and drives neurogenesis. J. Clin. Invest. 115, 2083–2098. doi: 10.1172/JCI23559, PMID: 16007267 PMC1172228

[ref122] Schulz C. Gomez Perdiguero E. Chorro L. Szabo-Rogers H. Cagnard N. Kierdorf K.. (2012). A lineage of myeloid cells independent of Myb and hematopoietic stem cells. Science 336, 86–90. doi: 10.1126/science.1219179, PMID: 22442384

[ref123] Smith M. E. (1993). Phagocytosis of myelin by microglia in vitro. J. Neurosci. Res. 35, 480–487. doi: 10.1002/jnr.490350504, PMID: 7690856

[ref124] Smith M. E. Van Der Maesen K. Somera F. P. (1998). Macrophage and microglial responses to cytokines *in vitro*: phagocytic activity, proteolytic enzyme release, and free radical production. J. Neurosci. Res. 54, 68–78. doi: 10.1002/(SICI)1097-4547(19981001)54:1<68::AID-JNR8>3.0.CO;2-F, PMID: 9778151

[ref125] Suh H. S. Kim M. O. Lee S. C. (2005). Inhibition of granulocyte-macrophage colony-stimulating factor signaling and microglial proliferation by anti-Cd45ro: role of Hck tyrosine kinase and phosphatidylinositol 3-kinase/Akt. J. Immunol. 174, 2712–2719. doi: 10.4049/jimmunol.174.5.2712, PMID: 15728479

[ref126] Sundal C. Baker M. Karrenbauer V. Gustavsen M. Bedri S. Glaser A.. (2015). Hereditary diffuse leukoencephalopathy with spheroids with phenotype of primary progressive multiple sclerosis. Eur. J. Neurol. 22, 328–333. doi: 10.1111/ene.12572, PMID: 25311247 PMC4289423

[ref127] Suzumura A. Sawada M. Yamamoto H. Marunouchi T. (1990). Effects of colony stimulating factors on isolated microglia in vitro. J. Neuroimmunol. 30, 111–120. doi: 10.1016/0165-5728(90)90094-4, PMID: 2229405

[ref128] Tambuyzer B. R. Nouwen E. J. (2005). Inhibition of microglia multinucleated giant cell formation and induction of differentiation by gm-Csf using a porcine in vitro model. Cytokine 31, 270–279. doi: 10.1016/j.cyto.2005.05.006, PMID: 16009563

[ref129] Tanaka J. Fujita H. Matsuda S. Toku K. Sakanaka M. Maeda N. (1997). Glucocorticoid- and mineralocorticoid receptors in microglial cells: the two receptors mediate differential effects of corticosteroids. Glia 20, 23–37. doi: 10.1002/(SICI)1098-1136(199705)20:1<23::AID-GLIA3>3.0.CO;2-6, PMID: 9145302

[ref130] Tarkowski E. Wallin A. Regland B. Blennow K. Tarkowski A. (2001). Local and systemic gm-Csf increase in Alzheimer's disease and vascular dementia. Acta Neurol. Scand. 103, 166–174. doi: 10.1034/j.1600-0404.2001.103003166.x, PMID: 11240564

[ref131] Thery C. Mallat M. (1993). Influence of interleukin-1 and tumor necrosis factor alpha on the growth of microglial cells in primary cultures of mouse cerebral cortex: involvement of colony-stimulating factor 1. Neurosci. Lett. 150, 195–199. doi: 10.1016/0304-3940(93)90534-R, PMID: 8469420

[ref132] Tian W. T. Zhan F. X. Liu Q. Luan X. H. Zhang C. Shang L.. (2019). Clinicopathologic characterization and abnormal autophagy of Csf1R-related leukoencephalopathy. Trans. Neurodegener. 8:32. doi: 10.1186/s40035-019-0171-y, PMID: 31827782 PMC6886209

[ref133] Timmerman R. Zuiderwijk-Sick E. A. Bajramovic J. J. (2022). P2Y6 receptor-mediated signaling amplifies Tlr-induced pro-inflammatory responses in microglia. Front. Immunol. 13:967951. doi: 10.3389/fimmu.2022.967951, PMID: 36203578 PMC9531012

[ref134] Tipton P. W. Stanley E. R. Chitu V. Wszolek Z. K. (2021). Is pre-symptomatic immunosuppression protective in Csf1R-related leukoencephalopathy? Mov. Disord. 36, 852–856. doi: 10.1002/mds.28515, PMID: 33590562 PMC8547119

[ref135] Tushinski R. J. Oliver I. T. Guilbert L. J. Tynan P. W. Warner J. R. Stanley E. R. (1982). Survival of mononuclear phagocytes depends on a lineage-specific growth factor that the differentiated cells selectively destroy. Cells 28, 71–81. doi: 10.1016/0092-8674(82)90376-2, PMID: 6978185

[ref136] Ueno M. Fujita Y. Tanaka T. Nakamura Y. Kikuta J. Ishii M.. (2013). Layer V cortical neurons require microglial support for survival during postnatal development. Nat. Neurosci. 16, 543–551. doi: 10.1038/nn.3358, PMID: 23525041

[ref137] Wake H. Moorhouse A. J. Miyamoto A. Nabekura J. (2013). Microglia: actively surveying and shaping neuronal circuit structure and function. Trends Neurosci. 36, 209–217. doi: 10.1016/j.tins.2012.11.007, PMID: 23260014

[ref138] Walker D. G. Tang T. M. Lue L. F. (2017). Studies on Colony stimulating factor Receptor-1 and ligands Colony stimulating Factor-1 and Interleukin-34 in Alzheimer's disease brains and human microglia. Front. Aging Neurosci. 9:244. doi: 10.3389/fnagi.2017.00244, PMID: 28848420 PMC5552759

[ref139] Wang Y. Berezovska O. Fedoroff S. (1999). Expression of colony stimulating factor-1 receptor (Csf-1R) by Cns neurons in mice. J. Neurosci. Res. 57, 616–632. doi: 10.1002/(SICI)1097-4547(19990901)57:5<616::AID-JNR4>3.0.CO;2-E, PMID: 10462686

[ref140] Wang H. Cao J. Lai X. (2016). Serum Interleukin-34 levels are elevated in patients with systemic lupus erythematosus. Molecules 22:35. doi: 10.3390/molecules22010035, PMID: 28036035 PMC6155597

[ref141] Wang S. Lai X. Deng Y. Song Y. (2020). Correlation between mouse age and human age in anti-tumor research: significance and method establishment. Life Sci. 242:117242. doi: 10.1016/j.lfs.2019.117242, PMID: 31891723

[ref142] Wang Y. Szretter K. J. Vermi W. Gilfillan S. Rossini C. Cella M.. (2012). Il-34 is a tissue-restricted ligand of Csf1R required for the development of Langerhans cells and microglia. Nat. Immunol. 13, 753–760. doi: 10.1038/ni.2360, PMID: 22729249 PMC3941469

[ref143] Wegiel J. Wisniewski H. M. Dziewiatkowski J. Tarnawski M. Kozielski R. Trenkner E.. (1998). Reduced number and altered morphology of microglial cells in colony stimulating factor-1-deficient osteopetrotic op/op mice. Brain Res. 804, 135–139. doi: 10.1016/S0006-8993(98)00618-0, PMID: 9729335

[ref144] Wei R. Jonakait G. M. (1999). Neurotrophins and the anti-inflammatory agents interleukin-4 (Il-4), Il-10, Il-11 and transforming growth factor-beta1 (Tgf-beta1) down-regulate T cell costimulatory molecules B7 and Cd40 on cultured rat microglia. J. Neuroimmunol. 95, 8–18. doi: 10.1016/S0165-5728(98)00248-3, PMID: 10229111

[ref145] Wei S. Nandi S. Chitu V. Yeung Y. G. Yu W. Huang M.. (2010). Functional overlap but differential expression of Csf-1 and Il-34 in their Csf-1 receptor-mediated regulation of myeloid cells. J. Leukoc. Biol. 88, 495–505. doi: 10.1189/jlb.1209822, PMID: 20504948 PMC2924605

[ref146] Werner K. Bitsch A. Bunkowski S. Hemmerlein B. Bruck W. (2002). The relative number of macrophages/microglia expressing macrophage colony-stimulating factor and its receptor decreases in multiple sclerosis lesions. Glia 40, 121–129. doi: 10.1002/glia.10120, PMID: 12237849

[ref147] Witt K. A. Sandoval K. E. (2014). Steroids and the blood-brain barrier: therapeutic implications. Adv. Pharmacol. 71, 361–390. doi: 10.1016/bs.apha.2014.06.018 25307223

[ref148] Xiao B. G. Xu L. Y. Yang J. S. (2002). Tgf-beta 1 synergizes with gm-Csf to promote the generation of glial cell-derived dendriform cells in vitro. Brain Behav. Immun. 16, 685–697. doi: 10.1016/S0889-1591(02)00020-X, PMID: 12480499

[ref149] Ximerakis M. Lipnick S. L. Innes B. T. Simmons S. K. Adiconis X. Dionne D.. (2019). Single-cell transcriptomic profiling of the aging mouse brain. Nat. Neurosci. 22, 1696–1708. doi: 10.1038/s41593-019-0491-3, PMID: 31551601

[ref150] Yamasaki R. Tanaka M. Fukunaga M. Tateishi T. Kikuchi H. Motomura K.. (2010). Restoration of microglial function by granulocyte-colony stimulating factor in Als model mice. J. Neuroimmunol. 229, 51–62. doi: 10.1016/j.jneuroim.2010.07.002, PMID: 20659772

[ref151] Yang T. T. Lin C. Hsu C. T. Wang T. F. Ke F. Y. Kuo Y. M. (2013). Differential distribution and activation of microglia in the brain of male C57bl/6J mice. Brain Struct. Funct. 218, 1051–1060. doi: 10.1007/s00429-012-0446-x, PMID: 22886465

[ref152] Yoshio T. Okamoto H. Kurasawa K. Dei Y. Hirohata S. Minota S. (2016). Il-6, Il-8, Ip-10, Mcp-1 and G-Csf are significantly increased in cerebrospinal fluid but not in sera of patients with central neuropsychiatric lupus erythematosus. Lupus 25, 997–1003. doi: 10.1177/0961203316629556, PMID: 26846690

[ref153] Yuan L. Liu A. Qiao L. Sheng B. Xu M. Li W.. (2015). The relationship of Csf and plasma cytokine levels in Hiv infected patients with neurocognitive impairment. Biomed. Res. Int. 2015:506872. doi: 10.1155/2015/506872, PMID: 25821806 PMC4363531

[ref154] Zhan L. Fan L. Kodama L. Sohn P. D. Wong M. Y. Mousa G. A.. (2020). A Mac2-positive progenitor-like microglial population is resistant to Csf1R inhibition in adult mouse brain. elife 9:e51796. doi: 10.7554/eLife.51796, PMID: 33054973 PMC7591254

[ref155] Zhao L. R. Navalitloha Y. Singhal S. Mehta J. Piao C. S. Guo W. P.. (2007). Hematopoietic growth factors pass through the blood-brain barrier in intact rats. Exp. Neurol. 204, 569–573. doi: 10.1016/j.expneurol.2006.12.001, PMID: 17307165 PMC3099460

[ref156] Zhu F. Liu Y. Zhao J. Zheng Y. (2014). Minocycline alleviates behavioral deficits and inhibits microglial activation induced by intrahippocampal administration of granulocyte-macrophage Colony-stimulating factor in adult rats. Neuroscience 266, 275–281. doi: 10.1016/j.neuroscience.2014.01.021, PMID: 24486961

